# Association of COVID-19 Vaccination With SARS-CoV-2 Infection in Patients With Cancer: A US Nationwide Veterans Affairs Study

**DOI:** 10.1001/jamaoncol.2021.5771

**Published:** 2021-12-02

**Authors:** Julie Tsu-Yu Wu, Jennifer La, Westyn Branch-Elliman, Linden B. Huhmann, Summer S. Han, Giovanni Parmigiani, David P. Tuck, Mary T. Brophy, Nhan V. Do, Albert Y. Lin, Nikhil C. Munshi, Nathanael R. Fillmore

**Affiliations:** 1 VA Palo Alto Healthcare System, Palo Alto, California; 2 Stanford University School of Medicine, Stanford, California; 3 VA Cooperative Studies Program, VA Boston Healthcare System, Boston, Massachusetts; 4 VA Boston Healthcare System, Section of Infectious Diseases, Boston, Massachusetts; 5 VA Boston Center for Healthcare Organization and Implementation Research (CHOIR), Boston, Massachusetts; 6 Harvard Medical School, Boston, Massachusetts; 7 Dana-Farber Cancer Institute, Boston, Massachusetts; 8 Harvard School of Public Health, Boston, Massachusetts; 9 VA Boston Healthcare System, Hematology/Oncology Service, Boston, Massachusetts; 10 Boston University School of Medicine, Boston, Massachusetts

## Abstract

**Importance:**

Patients with cancer are at increased risk for severe COVID-19, but it is unknown whether SARS-CoV-2 vaccination is effective for them.

**Objective:**

To determine the association between SARS-CoV-2 vaccination and SARS-CoV-2 infections among a population of Veterans Affairs (VA) patients with cancer.

**Design, Setting, and Participants:**

Retrospective, multicenter, nationwide cohort study of SARS-CoV-2 vaccination and infection among patients in the VA health care system from December 15, 2020, to May 4, 2021. All adults with solid tumors or hematologic cancer who received systemic cancer-directed therapy from August 15, 2010, to May 4, 2021, and were alive and without a documented SARS-CoV-2 positive result as of December 15, 2020, were eligible for inclusion. Each day between December 15, 2020, and May 4, 2021, newly vaccinated patients were matched 1:1 with unvaccinated or not yet vaccinated controls based on age, race and ethnicity, VA facility, rurality of home address, cancer type, and treatment type/timing.

**Exposures:**

Receipt of a SARS-CoV-2 vaccine.

**Main Outcomes and Measures:**

The primary outcome was documented SARS-CoV-2 infection. A proxy for vaccine effectiveness was defined as 1 minus the risk ratio of SARS-CoV-2 infection for vaccinated individuals compared with unvaccinated controls.

**Results:**

A total of 184 485 patients met eligibility criteria, and 113 796 were vaccinated. Of these, 29 152 vaccinated patients (median [IQR] age, 74.1 [70.2-79.3] years; 95% were men; 71% were non-Hispanic White individuals) were matched 1:1 to unvaccinated or not yet vaccinated controls. As of a median 47 days of follow-up, 436 SARS-CoV-2 infections were detected in the matched cohort (161 infections in vaccinated patients vs 275 in unvaccinated patients). There were 17 COVID-19–related deaths in the vaccinated group vs 27 COVID-19–related deaths in the unvaccinated group. Overall vaccine effectiveness in the matched cohort was 58% (95% CI, 39% to 72%) starting 14 days after the second dose. Patients who received chemotherapy within 3 months prior to the first vaccination dose were estimated to have a vaccine effectiveness of 57% (95% CI, –23% to 90%) starting 14 days after the second dose vs 76% (95% CI, 50% to 91%) for those receiving endocrine therapy and 85% (95% CI, 29% to 100%) for those who had not received systemic therapy for at least 6 months prior.

**Conclusions and Relevance:**

In this cohort study, COVID-19 vaccination was associated with lower SARS-CoV-2 infection rates in patients with cancer. Some immunosuppressed subgroups may remain at early risk for COVID-19 despite vaccination, and consideration should be given to additional risk reduction strategies, such as serologic testing for vaccine response and a third vaccine dose to optimize outcomes.

## Key Points

**Question:**

What is the effectiveness of SARS-CoV-2 vaccination in patients with cancer?

**Findings:**

In this cohort study of US Veterans Affairs patients who received systemic therapy for cancer between August 15, 2010, and May 4, 2021, a proxy measure for effectiveness of the vaccine starting 14 days after the second dose was 58%. The measure of effectiveness starting 14 days after the second dose was 85% in patients who had not received systemic therapy within the 6 months prior to vaccination and 76% among those receiving hormonal treatment.

**Meaning:**

Results suggest that SARS-CoV-2 vaccination associated with lower infection rates in patients with cancer, especially in those not receiving current systemic therapy and those receiving hormonal treatment.

## Teaser

This cohort study assesses the association between SARS-CoV-2 vaccination and SARS-CoV-2 infections among a population of Veterans Affairs (VA) patients with cancer.

## Introduction

Although patients with cancer have poor COVID-19–related outcomes,^1,2,3,4^ the effectiveness of SARS-CoV-2 vaccination in this population is unclear because they were excluded from vaccination trials.^5,6^ Recent studies suggest that patients with cancer mount less robust antibody responses to vaccination than immunocompetent controls.^7,8,9,10^ The objective of this retrospective cohort study in the national Veterans Affairs (VA) health care system was to estimate the SARS-CoV-2 vaccination effectiveness in patients with cancer in a real-world setting during the 140-day period following initial vaccine availability.

## Methods

### Study Population and Data Sources

Patients were included if they were administered cancer-directed therapy within 7 days before or after a solid tumor or hematologic cancer *International Statistical Classification of Diseases and Related Health Problems, Tenth Revision (ICD-10)* code during August 15, 2010, to May 4, 2021, and were alive without documented SARS-CoV-2 infection prior to vaccine availability on December 15, 2020 (eFigure 1 in the Supplement). Patients were excluded if they were vaccinated with Janssen/Johnson & Johnson Ad26.COV2.S or were not regular VA users (eMethods in the Supplement). This study was approved by the VA Boston Healthcare System Research and Development Committee and received a waiver of informed consent because the study presented minimal risk and could not practicably be conducted without a waiver.

### Matching and Cohort Creation

Vaccinated patients were matched 1:1 to unvaccinated controls using a previously described approach designed to emulate a randomized clinical trial.^11^ The purpose of this procedure is to reduce bias by matching vaccinated patients to unvaccinated patients who had a similar probability of vaccination and SARS-CoV-2 exposure but had not yet been vaccinated.

For each day from December 15, 2020, to May 4, 2021, every newly vaccinated patient was matched with a patient not yet vaccinated. Patients who had a positive SARS-CoV-2 test result prior to vaccination date or matching as control were excluded. Follow-up time was censored by the earliest of the following: vaccination (for unvaccinated controls), vaccination of the matched control (for vaccinated patients), end of study period, or death.

Matching was based on factors potentially associated with SARS-CoV-2 vaccination and exposure to SARS-CoV-2, specifically age, race and ethnicity, VA facility, rurality of home address, cancer type, and treatment type/timing. We used minimum distance matching for age and exact matching for the others (see eMethods, eFigure 6, and eTable 13 in the Supplement for matching algorithm and variable definition details).

### Outcomes

The primary outcome was reverse transcriptase–polymerase chain reaction or antigen-confirmed SARS-CoV-2 infection. Secondary outcomes included COVID-19–related death, or death within 4 weeks of COVID-19 diagnosis (eMethods in the Supplement). A proxy measure of vaccine effectiveness was defined as 1 minus the risk ratio of SARS-CoV-2 infection for vaccinated individuals compared with unvaccinated controls. For example, if there were 10 cases out of 100 among the unvaccinated, a vaccine effectiveness of 90% would mean there was 1 case among the vaccinated. The Fine-Gray subdistribution hazard function was used to measure risk. Statistical analysis is detailed in eMethods in the Supplement. Subgroup analyses were determined a priori based on potential association with vaccine effectiveness: solid tumor vs hematologic cancer, time from last treatment, and type of treatment received.

## Results

Among 184 485 eligible patients, 113 796 (62%) were vaccinated during the study period (eFigure 1 in the Supplement). Of these, 29 152 vaccinated patients (median [IQR] age, 74.1 [70.2-79.3] years; 95% were men; 71% were non-Hispanic White individuals) were matched 1:1 to 29 152 unvaccinated controls. Among 109 338 patients who received the first vaccine dose and had at least 28 days of follow-up, 106 109 (97%) received a second dose. For analysis of second dose vaccine effectiveness, 18 312 pairs were included. Baseline demographics of the unmatched and matched cohorts are shown in eTables 1 and 2 in the Supplement.

As of a median 47 days of follow-up, 436 SARS-CoV-2 infections were detected in the matched cohort (161 infections in vaccinated patients vs 275 in unvaccinated patients). There were 17 COVID-19–related deaths in the vaccinated group vs 27 COVID-19–related deaths in the unvaccinated group.

**Figure 1.**
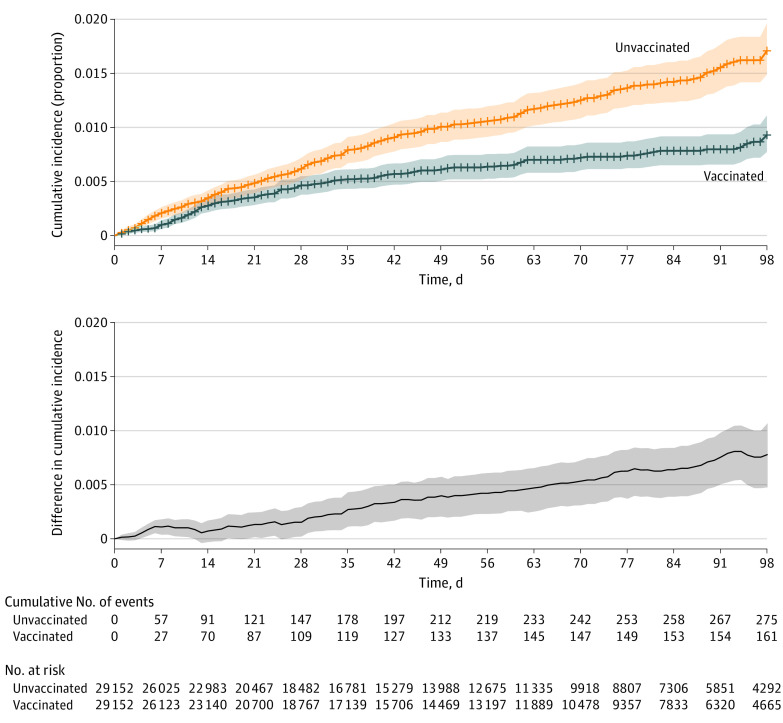
Cumulative Incidence of SARS-CoV-2 Infection After First Vaccine Dose in the Overall Matched Cohort Cumulative incidence curves of SARS-CoV-2 infection in the overall matched cohort using time zero as the date of the first dose of vaccination. The difference in cumulative incidence is illustrated in the gray bottom graph. Shaded areas indicate 95% CIs calculated by bootstrapping.

Figure 1 shows cumulative incidence of SARS-CoV-2 infection in the full matched cohort. Estimated overall vaccine effectiveness following first dose was 42% (95% CI, 28% to 53%). The difference in infections between the vaccinated and unvaccinated cohorts remained near zero in the 2 weeks following the first dose, when immunity is building. Both messenger RNA vaccines had similar effectiveness (eFigure 2 in the Supplement).

The Table shows estimated vaccine effectiveness in the period starting 14 days after the second dose in the overall cohort and predetermined subgroups (additional time periods in eTable 3 in the Supplement). Overall effectiveness in this period was 58% (95% CI, 39% to 73%). Effectiveness estimates were higher among patients with solid tumors (effectiveness starting 14 days after the second dose, 66%; 95% CI, 48% to 79%) than hematologic cancers (19%; 95% CI, −68% to 65%) (eFigure 3, eTables 4 and 5 in the Supplement). Patients who last received systemic therapy more than 6 months before vaccination exhibited effectiveness of 85% starting 14 days after the second dose (95% CI, 29% to 100%), vs 63% (95% CI, 23% to 87%) among patients whose last therapy was 3 to 6 months prior and 54% (95% CI, 28% to 72%) in patients who received therapy within 3 months prior (eFigure 4, eTables 6-9 in the Supplement).

**Table.** Estimated Vaccine Effectiveness Against SARS-CoV-2 Infection Starting 14 Days After the Second Dose in Overall Cohort and Subgroups

Group	1 − RR (95% CI)
Overall	58 (39 to 73)
Cancer category	
Solid malignant neoplasm	66 (48 to 79)
Hematologic cancer	19 (−68 to 65)
Treatment timing^a^	
Distant treatment (>6 mo)	85 (29 to 100)
Recent treatment (3-6 mo)	63 (23 to 87)
Current treatment (0-3 mo)	54 (28 to 72)
Treatment after vaccine	49 (–110 to 100)
Treatment type (0-3 mo)^b^	
Current chemotherapy containing	57 (−23 to 91)
Current targeted	29 (−84 to 75)
Current endocrine	76 (50 to 91)

^a^ Timing of systemic therapy relative to the date of vaccination (if in the vaccinated cohort) or entry date (if in the unvaccinated cohort), as detailed in eMethods in the Supplement.

^b^ Systemic therapy split by treatment type received in the 3 months prior to vaccination (if in the vaccinated cohort) or prior to entry date (if in the unvaccinated cohort). If any chemotherapy agent is administered in combination with another agent (eg, chemoimmunotherapy), it was considered a chemotherapy-containing regimen. Treatment type determination is further detailed in eMethods in the Supplement. Estimation of vaccine effectiveness in patients receiving immunotherapy was not feasible owing to inadequate number of events.

**Figure 2.**
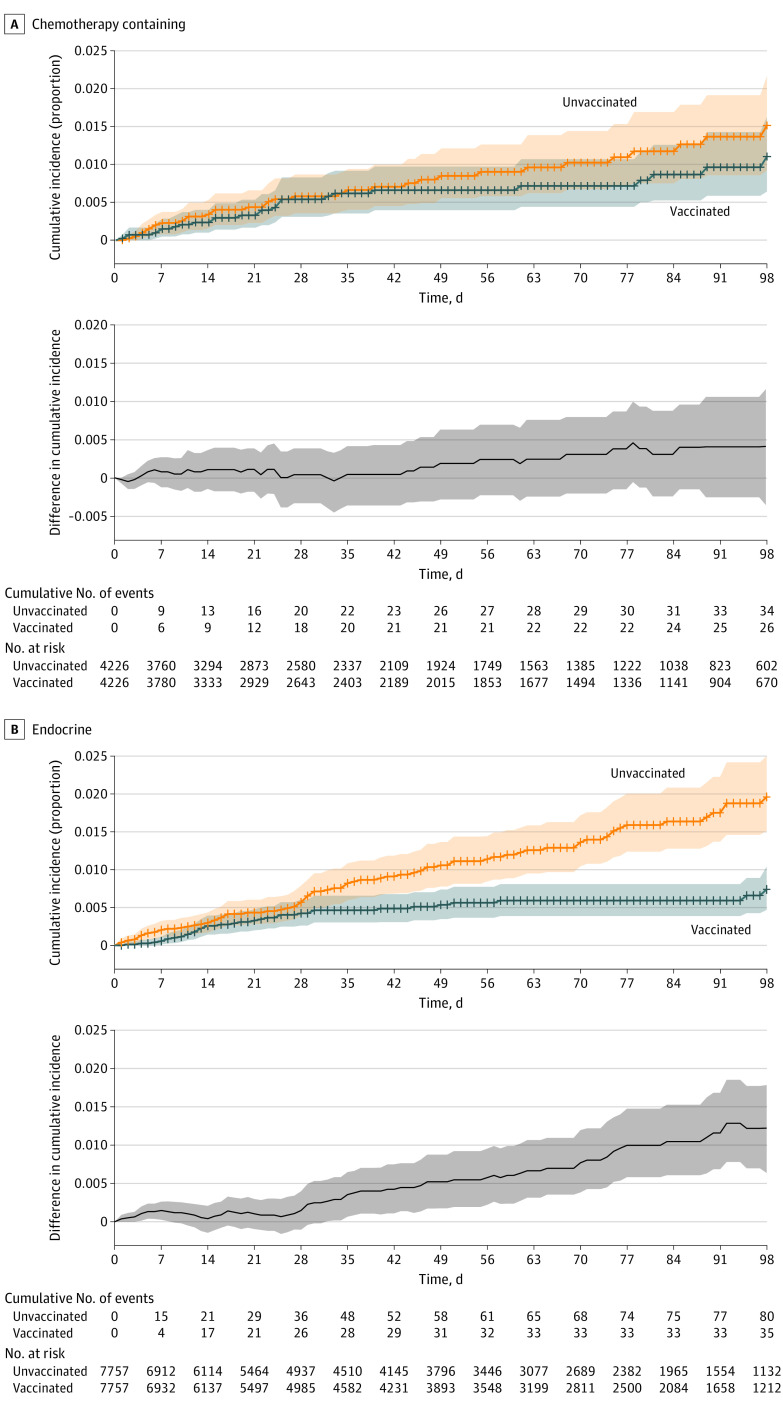
Cumulative Incidence of SARS-CoV-2 Infection After First Vaccine Dose in Patients Receiving Current Treatment With Chemotherapy-Containing Regimens and Endocrine Therapy Cumulative incidence curves of SARS-CoV-2 infection among patients receiving current treatment with (A) a chemotherapy-containing regimen and (B) an endocrine therapy regimen at the time of vaccination. Time zero is the date of the first dose of vaccination or entry as an unvaccinated control. The difference in cumulative incidence is illustrated in the gray bottom graphs. Shaded areas indicate 95% CIs calculated by bootstrapping. Current treatment is defined as systemic therapy received within the 3 months prior to vaccination (if in the vaccinated group) or prior to entry date (if in the unvaccinated group).

Figure 2 shows the cumulative incidence of SARS-CoV-2 infection in patients who received chemotherapy-containing regimens vs endocrine therapy within 3 months before vaccination (additional treatment types in eFigure 5 in the Supplement). Vaccination appears less effective for patients receiving chemotherapy (effectiveness starting 14 days after the second dose, 57%; 95% CI, –23% to 91%) vs endocrine therapy (76%; 95% CI, 50% to 91%) (eTables 10-12 in the Supplement).

## Discussion

Vaccination in VA patients with cancer is effective at reducing the incidence of SARS-CoV-2 infection. Fewer COVID-19–related deaths were observed in vaccinated than in unvaccinated patients, although estimation of vaccine effectiveness for death is limited by low event rates. Our study is the first, to our knowledge, to demonstrate vaccine effectiveness against SARS-CoV-2 infection in patients with cancer.

Vaccine effectiveness may decrease with age,^11,12^ and malignancy and anticancer treatments may be immunosuppressive.^7,8,9,10,13,14^ These factors may account for the reduced effectiveness we observe among some patients with cancer vs effectiveness reported in the general population recently estimated to be 89% to 92%.^11,12^ Notably, in our study, vaccine effectiveness in patients receiving endocrine therapy and those last receiving therapy more than 6 months before vaccination was similar to general population estimates. We independently identify previously demonstrated risk factors for poor vaccination response. Hematologic cancers and immunosuppressive therapies are associated with reduced antibody response to vaccination^7,8,9,10,13,14^ and lower effectiveness in our study. Our findings suggest that high-risk populations may benefit from additional mitigation measures, such as extended masking. Data from other immunosuppressed populations suggest that a third vaccine dose may improve effectiveness^15^; additional work is needed to determine efficacy of a similar approach in patients with cancer, particularly those who were vaccinated during active treatment.

### Limitations

This study has limitations. Owing to limited power, we cannot robustly assess vaccination effectiveness against severe COVID-19 and COVID-19–related deaths. However, we found a reduction in SARS-CoV-2 infections, suggesting that vaccines mitigate against these important outcomes. An in-study comparison of patients with cancer with patients without cancer is not possible because the latter were not included. Because veterans may receive care outside the VA, exposure to vaccination, SARS-CoV-2, and covariates may be misclassified. However, the cohort included only patients receiving cancer care within the VA to reduce potential for missing data and matched on a wide range of factors associated with likelihood of vaccination and SARS-CoV-2 exposure. Reassuringly, the matched vaccinated and unvaccinated cohorts demonstrated similar rates of infection until day 14, when antibody-mediated immunity begins. This early period serves as a negative control that demonstrates the robustness of our matching.

## Conclusions

In this cohort study, COVID-19 vaccination was associated with lower risk of SARS-CoV-2 infections in patients with cancer in a real-world setting. Additional studies including a different patient population with longer follow-up period will be valuable, as will studies to delineate optimal vaccination regimen and administration relative to cancer and treatment type.

## Notes

Supplement.
eMethods.
**eFigure 1.** Flow Diagram of Cohort Selection and Matching. **eFigure 2.** Cumulative Incidence and Difference in SARS-COV-2 Infection between Matched Vaccinated and Unvaccinated Cohorts Stratified by Vaccination Type. **eFigure 3.** Cumulative Incidence and Difference in SARS-COV-2 Infection between Matched Vaccinated and Unvaccinated Cohorts Stratified by Cancer Category. **eFigure 4.** Cumulative Incidence and Difference in SARS-COV-2 Infection between Matched Vaccinated and Unvaccinated Cohorts Stratified by Treatment Timing. **eFigure 5.** Cumulative Incidence and Difference in SARS-COV-2 Infection between Matched Vaccinated and Unvaccinated Cohorts Stratified by Additional Treatment Types. **eFigure 6.** Cancer Type Determination Algorithm. **eTable 1.** Demographic and Clinical Characteristics of Vaccinated and Unvaccinated VA Cancer Patients in the Pre-match Cohort. **eTable 2. **Demographic and Clinical Characteristics of Vaccinated and Unvaccinated VA Cancer Patients in the Matched Cohort. **eTable 3.** Estimated Vaccine Effectiveness against SARS-COV-2 Infection with Alternate Time Periods. **eTable 4.** Demographic and Clinical Characteristics of Matched Vaccinated and Unvaccinated VA Cancer Patients with Solid Malignancies. **eTable 5.** Demographic and Clinical Characteristics of Matched Vaccinated and Unvaccinated VA Cancer Patients with Hematologic Malignancies. **eTable 6.** Demographic and Clinical Characteristics of Matched Vaccinated and Unvaccinated VA Cancer Patients with Distant Treatment (>6 Months). **eTable 7.** Demographic and Clinical Characteristics of Matched Vaccinated and Unvaccinated VA Cancer Patients with Recent Treatment (3-6 Months). **eTable 8.** Demographic and Clinical Characteristics of Matched Vaccinated and Unvaccinated VA Cancer Patients with Current Treatment (<3 Months). **eTable 9.** Demographic and Clinical Characteristics of Matched Vaccinated and Unvaccinated VA Cancer Patients with Treatment After Vaccination. **eTable 10.** Demographic and Clinical Characteristics of Matched Vaccinated and Unvaccinated VA Cancer Patients on Current Chemotherapy-containing Treatment (<3 Months). **eTable 11.** Demographic and Clinical Characteristics of Matched Vaccinated and Unvaccinated VA Cancer Patients on Current Targeted Treatment (<3 Months). **eTable 12.** Demographic and Clinical Characteristics of Matched Vaccinated and Unvaccinated VA Cancer Patients on Current Endocrine Treatment (<3 Months). **eTable 13.** Cancer-directed Systemic Therapies by Treatment Type.
