# Supplementary material for: Association of COVID-19 Vaccination With SARS-CoV-2 Infection in Patients With Cancer: A US Nationwide Veterans Affairs Study
Source: JAMA Oncol. 2021 Dec 2;8(2):1–6. doi: 10.1001/jamaoncol.2021.5771 (PMC8640949; doi:10.1001/jamaoncol.2021.5771)
Supplement: Supplement. — eMethods. eFigure 1. Flow Diagram of Cohort Selection and Matching. eFigure 2. Cumulative Incidence and Difference in SARS-COV-2 Infection between Matched Vaccinated and Unvaccinated Cohorts Stratified by Vaccination Type. eFigure 3. Cumulative Incidence and Difference in SARS-COV-2 Infection between Matched Vaccinated and Unvaccinated Cohorts Stratified by Cancer Category. eFigure 4. Cumulative Incidence and Difference in SARS-COV-2 Infection between Matched Vaccinated and Unvaccinated Cohorts Stratified by Treatment Timing. eFigure 5. Cumulative Incidence and Difference in SARS-COV-2 Infection between Matched Vaccinated and Unvaccinated Cohorts Stratified by Additional Treatment Types. eFigure 6. Cancer Type Determination Algorithm. eTable 1. Demographic and Clinical Characteristics of Vaccinated and Unvaccinated VA Cancer Patients in the Pre-match Cohort. eTable 2. Demographic and Clinical Characteristics of Vaccinated and Unvaccinated VA Cancer Patients in the Matched Cohort. eTable 3. Estimated Vaccine Effectiveness against SARS-COV-2 Infection with Alternate Time Periods. eTable 4. Demographic and Clinical Characteristics of Matched Vaccinated and Unvaccinated VA Cancer Patients with Solid Malignancies. eTable 5. Demographic and Clinical Characteristics of Matched Vaccinated and Unvaccinated VA Cancer Patients with Hematologic Malignancies. eTable 6. Demographic and Clinical Characteristics of Matched Vaccinated and Unvaccinated VA Cancer Patients with Distant Treatment (>6 Months). eTable 7. Demographic and Clinical Characteristics of Matched Vaccinated and Unvaccinated VA Cancer Patients with Recent Treatment (3-6 Months). eTable 8. Demographic and Clinical Characteristics of Matched Vaccinated and Unvaccinated VA Cancer Patients with Current Treatment (<3 Months). eTable 9. Demographic and Clinical Characteristics of Matched Vaccinated and Unvaccinated VA Cancer Patients with Treatment After Vaccination. eTable 10. Demographic and Clinical Characteristics o [file jamaoncol-e215771-s001.pdf]

Supplemental Online Content

Wu JT-Y, La J, Branch-Elliman W, et al. Association of COVID-19 vaccination with SARS-CoV-2 infection in patients with cancer: a US nationwide Veterans Affairs study. *JAMA Oncol*. Published online December 2, 2021. doi:10.1001/jamaoncol.2021.5771

eMethods.

eFigure 1. Flow Diagram of Cohort Selection and Matching.

eFigure 2. Cumulative Incidence and Difference in SARS-COV-2 Infection between Matched Vaccinated and Unvaccinated Cohorts Stratified by Vaccination Type.

eFigure 3. Cumulative Incidence and Difference in SARS-COV-2 Infection between Matched Vaccinated and Unvaccinated Cohorts Stratified by Cancer Category.

eFigure 4. Cumulative Incidence and Difference in SARS-COV-2 Infection between Matched Vaccinated and Unvaccinated Cohorts Stratified by Treatment Timing.

eFigure 5. Cumulative Incidence and Difference in SARS-COV-2 Infection between Matched Vaccinated and Unvaccinated Cohorts Stratified by Additional Treatment Types.

eFigure 6. Cancer Type Determination Algorithm.

eTable 1. Demographic and Clinical Characteristics of Vaccinated and Unvaccinated VA Cancer Patients in the Pre-match Cohort.

eTable 2. Demographic and Clinical Characteristics of Vaccinated and Unvaccinated VA Cancer Patients in the Matched Cohort.

eTable 3. Estimated Vaccine Effectiveness against SARS-COV-2 Infection with Alternate Time Periods.

eTable 4. Demographic and Clinical Characteristics of Matched Vaccinated and Unvaccinated VA Cancer Patients with Solid Malignancies.

eTable 5. Demographic and Clinical Characteristics of Matched Vaccinated and Unvaccinated VA Cancer Patients with Hematologic Malignancies.

eTable 6. Demographic and Clinical Characteristics of Matched Vaccinated and Unvaccinated VA Cancer Patients with Distant Treatment (>6 Months).

eTable 7. Demographic and Clinical Characteristics of Matched Vaccinated and Unvaccinated VA Cancer Patients with Recent Treatment (3-6 Months).

eTable 8. Demographic and Clinical Characteristics of Matched Vaccinated and Unvaccinated VA Cancer Patients with Current Treatment (<3 Months).

eTable 9. Demographic and Clinical Characteristics of Matched Vaccinated and Unvaccinated VA Cancer Patients with Treatment After Vaccination.

eTable 10. Demographic and Clinical Characteristics of Matched Vaccinated and Unvaccinated VA Cancer Patients on Current Chemotherapy-containing Treatment (<3 Months).

eTable 11. Demographic and Clinical Characteristics of Matched Vaccinated and Unvaccinated VA Cancer Patients on Current Targeted Treatment (<3 Months).

© 2021 Wu JTY et al. *JAMA Oncology.*

eTable 12. Demographic and Clinical Characteristics of Matched Vaccinated and Unvaccinated VA Cancer Patients on Current Endocrine Treatment (<3 Months).

eTable 13. Cancer-directed Systemic Therapies by Treatment Type.

This supplemental material has been provided by the authors to give readers additional information about their work.

© 2021 Wu JTY et al. *JAMA Oncology.*

eMethods

Data Sources

This study was primarily based on data in the VA Corporate Data Warehouse (CDW), which collates electronic health record (EHR) information from Veterans treated at VA facilities nationwide[1]. Information on SARS-CoV-2 vaccination and documented infection was obtained from the VA COVID-19 Shared Data Resource.

Variable Definitions

VA user was defined as patients with at least 1 ICD-10 code in each of the three years prior to 12/15/2020.

Documented SARS-CoV-2 infection and the date of first infection were based on structured information in the VA COVID-19 Shared Data Resource. Since all patients with infection prior to the beginning of study were excluded, only the date of first infection was of relevance.

Date of SARS-CoV-2 vaccination and vaccine type (Moderna, Pfizer, Janssen, or Unknown) were based on structured information in the VA COVID-19 Shared Data Resource.

Age was defined as the age on December 15, 2020 based on birth date recorded in the CDW.

Gender was based on structured data recorded in the CDW.

Race/ethnicity was based on self-reported race and ethnicity data as recorded in the CDW, with categories Hispanic (ethnicity "Hispanic or Latino"), Non-Hispanic White (ethnicity not "Hispanic or Latino", race "White"), Non-Hispanic Black (ethnicity not "Hispanic or Latino", race "Black or African American"), Other or Unknown (all others).

Rurality was defined based on a classification of each patient's home address as recorded in the CDW. The CDW distinguishes "rural" and "highly rural" categories, which we combined to reduce the number of categories for exact matching.

Geographical region was defined based on the VA facility each patient received cancer treatment in. Regions were defined using the following mapping from the CDW: Continental (Arkansas, Colorado, Louisiana, Mississippi, Montana, Oklahoma, Texas, Utah, Wyoming); Midwest (Illinois, Indiana, Michigan, Minnesota, Missouri, Nebraska, North Dakota, Ohio, South Dakota, Wisconsin); North Atlantic (Connecticut, Delaware, District Of Columbia, Maine, Maryland, Massachusetts, New Hampshire, New Jersey, New York, North Carolina, Pennsylvania, Rhode Island, Vermont, Virginia, West Virginia); Pacific (Alaska, Arizona, California, Hawaii, Idaho, Nevada, New Mexico, Oregon, Washington); Southeast (Alabama, Florida, Georgia, Kentucky, Puerto Rico, South Carolina, Tennessee).

The Charlson Comorbidity Index was calculated based on ICD-10 codes in the year prior to December 15, 2020 using the R comorbidity package[2].

Treatment timing prior to vaccination (if in the vaccinated cohort) or prior to entry date (if in the unvaccinated cohort) was defined based on pharmacy records in the CDW. Four treatment timing categories were defined: (1) Distant treatment; (2) Recent treatment; (3) Current treatment; (4) Treatment after vaccine. Regarding category (1), note that all patients in the study population received cancer-directed therapy on or after August 15, 2010, but this treatment ceased at least six months prior to the date of vaccination or matching entry date. The patients in category (2) had received their last dose of systemic therapy sometime in the period between three and six months prior to vaccination or matching. Patients in category (2) therefore represent a group that received systemic therapy more recently than the patients in category (1) but are no longer on active therapy at the time of vaccination or matching. Regarding category (3), patients were included if a patient received any systemic therapy within three months prior to vaccination or matching. Patients in category (4) received their first dose of systemic therapy after vaccination or matching but prior to study end date of May 4, 2021.

Treatment type was defined for the patients on current therapy at the time of vaccination (if in the vaccinated cohort) or entry date (if in the unvaccinated cohort). Patients were considered to be on current therapy if a patient received any systemic therapy within three months prior to vaccination or matching. All agents administered within the three month window were considered. Individual drugs were classified as

© 2021 Wu JTY et al. *JAMA Oncology.*

chemotherapy, immunotherapy, targeted therapy, or endocrine therapy using the mapping in eTable 13. If all the agents given to a patient in a three month period were the same type, that patient was assigned to that treatment type category. Regimens consisting of combinations of treatment types were prioritized in the following order: If any chemotherapy agent was administered in combination with another agent (e.g., chemoimmunotherapy), it was considered a chemotherapy-containing regimen. If immunotherapy agent was administered in combination with another agent that was not chemotherapy, it was considered an immunotherapy regimen. Targeted regimens contain targeted agents, but not chemotherapy or immunotherapy. Endocrine regimens were composed exclusively of endocrine therapy agents. All routes of cancer treatment were included, including oral, intravenous, intramuscular and subcutaneous.

Cancer type was determined from malignancy ICD-10 codes by the following algorithm for each patient (eFigure 6). For each patient, the most recent malignancy ICD-10 code(s) associated with a cancer-directed systemic treatment was identified. If there was a unique ICD-10 associated with that encounter, then the cancer type associated with that code was used. However, if there were multiple ICD-10 codes, and the ICD-10 codes were associated with different cancer types, ties were broken in with the following stepwise algorithm. If one of the ICD-10 codes were for malignancy of brain, bone, or lymph node, the primary site was assumed to be the remaining code(s) since brain, bone, and lymph nodes are common metastatic sites. For example, if a patient was identified by both a colorectal malignancy-associated ICD-10 code and a bone malignancy- associated ICD-10 code, the colorectal malignancy was assumed to be the primary site. If there were still a tie, the primary malignancy was decided by majority vote of the malignancy ICD-10 codes over the past 6 months prior to the most recent treatment-associated malignancy ICD-10 code. Remaining ties were broken via cancer type and treatment matching using a cancer type-treatment matrix. For example, if a patient received antiandrogen therapy and had a prostate malignancy-associated ICD-10 code, that patient was assumed to have prostate cancer. If the patient received a therapy associated with more than one cancer type, the patient was assumed to have the more common cancer type based on SEER national cancer prevalence statistics[3]. If a patient did not receive any of the typical treatments associated with their malignancy ICD-10 code, that patient was assigned to the other malignancy ICD-10 code. Remaining ties were classified as unknown.

Cancer category was determined using the cancer type as defined above. Hematologic malignancies were defined as essential thrombocythemia, leukemia/myelodysplastic syndrome (MDS)/myelofibrosis (MF), multiple myeloma, mastocytoma, and polycythemia vera. Solid malignancies were defined as brain, breast, colorectal, connective and soft tissue, squamous cell carcinoma of the head and neck (SCCHN)/cutaneous SCC (CSCC)/skin, esophagus/gastric, gynecologic malignancies (Gyn), hepatocellular carcinoma (HCC), lung, lymphoma, melanoma, neuroendocrine, anal/biliary/GIST/pancreas/small intestine (other GI), prostate, renal cell carcinoma (RCC), and urothelial cancer. ICD codes classified as "other/unknown" was split among the solid and hematologic malignancies based on the specific ICD code.

Total death was defined as any death recorded in the CDW occurring on the day of or in the four weeks following SARS-CoV-2 infection or a censoring event. Example: Patient 1, a vaccinated patient was vaccinated with their first dose on December 15th, 2020, infected with COVID-19 on January 1st 2021, and died on January 21st 2021. Patient 2, patient 1's unvaccinated matched control, was vaccinated on December 20th 2020. Given that patient 2 was vaccinated on December 20th 2020, both Patient 1 and Patient 2 would be censored on that day (Patient 2 censored as control and re-enters study as vaccinated). Although Patient 1 did indeed experience a death, this patient would not be counted in the total death because January 21st 2021 is more than 28 days past Patient 1's censored date. COVID-19 related death was defined as any death recorded in the CDW within four weeks after documentation of SARS-CoV-2 infection, excluding patients who were censored from the study population prior to infection[4]. In the example above, although Patient 1's death was within 28 days of a SARS-COV-2 infection, the death would not be counted as a COVID-19 related death because Patient 1 was censored. On the other hand, if Patient 2 (Patient 1's matched control) was vaccinated on January 5th 2021, then Patient 1's death would be counted as a COVID-19 related death.

Matching Algorithm

Vaccinated patients were matched with unvaccinated patients on predictors of SARS-COV-2 infection and vaccination. These variables included age, race/ethnicity, VA facility, rurality of home address, cancer type, and treatment type/timing. Age was matched with minimum distance matching and all other variables were matched with exact matching. So, a non-Hispanic white veteran with a rural home address and prostate cancer on endocrine therapy at the Boston VA will only be matched with another non-Hispanic white veteran with a rural home address and prostate cancer on endocrine therapy at the Boston VA. If more than one control patient matches the vaccinated patient, the control closest in age to the vaccinated patient is chosen. If there are no

© 2021 Wu JTY et al. *JAMA Oncology.*

controls available that exactly match the vaccinated patient, the unmatched vaccinated patient is discarded. Matching on additional variables (gender, comorbidity index, BMI) was not included, as their inclusion decreased sample size but did not improve matching quality.

During this matching process, patients who received vaccination after the matching date were eligible to be matched as an unvaccinated control. Excluding these patients would create a bias, since people who are symptomatic from early infection may avoid getting vaccinated and thereby bias the unvaccinated cohort towards a higher early COVID infection rate. If an unvaccinated control was subsequently vaccinated, follow-up for both the control and corresponding case was censored on the date of the control's vaccination. After the control's vaccination, the control would cross over to the vaccinated cohort and would be matched to a new unvaccinated control.

To assess for residual confounding, and thereby evaluate matching quality, as in the prior study[5] we examined the cumulative incidence of infection in the first 14 days of follow-up for both vaccinated and controls. This provides a "negative control" in regard to confounding. During this initial period, vaccinated and unvaccinated groups are expected to exhibit similar rates of infection if exchangeability between the groups holds, i.e., if there is no confounding.

Statistical Analysis

For first-dose effectiveness analyses, the index date for each vaccinated patient was set to their vaccination date, and the index date for their matched control was set to this same date. For analyses evaluating effectiveness after the second dose, date of second vaccination was used as the index date for each vaccinated patient and their matched control. For second-dose analyses, matched pairs where (1) the vaccinated patient did not have a second dose, (2) either member was infected before the second-dose index date, (3) the control died before the second-dose index date, or (4) the control was vaccinated before the second-dose index date were excluded.

Curves of cumulative incidence of infection for vaccinated patients and unvaccinated controls were generated using a Fine-Gray adjusted proportional subdistribution hazards model accounting for the competing risk of death[6], and adjusting for all variables used in matching except for VA facility and cancer type, as these variables introduced too many levels. The Fine-Gray subdistribution hazard function was used to measure risk. These measures were evaluated over pre-defined intervals analogous to those used in the original Pfizer BNT162b2 clinical trial[7]: day 0 after the first dose of vaccine to end of study, day 0 after the first dose to date of second dose, day 0 to day 13 after the second dose and day 14 after the second dose to end of follow-up. As protective immunity is expected to be minimal in the first two weeks after vaccination, a secondary analysis was performed considering the interval day 14 after the first dose to the date of the second dose. Confidence intervals for the effectiveness and cumulative incidence curves were calculated using percentile bootstrapping. Analyses were performed using R version 4.0.2.

© 2021 Wu JTY et al. *JAMA Oncology.*

References

1. Price LE, Shea K, Gephart S. The Veterans Affairs's Corporate Data Warehouse: Uses and Implications for Nursing Research and Practice. *Nurs Adm Q*. 2015;39(4):311-318. doi:10.1097/NAQ.0000000000000118

2. Gasparini A. comorbidity: An R package for computing comorbidity scores. *JOSS*. 2018;3(23):648. doi:10.21105/joss.00648

3. Henley SJ, Ward EM, Scott S, et al. Annual report to the nation on the status of cancer, part I: National cancer statistics. *Cancer*. 2020;126(10):2225-2249. doi:https://doi.org/10.1002/cncr.32802

4. Testa C, Krieger N, Chen J, Hanage W. Visualizing the lagged connection between COVID-19 cases and deaths in the United States: An animation using per capita state-level data (January 22, 2020 – July 8, 2020). Published online July 10, 2020. Accessed June 2, 2021. https://dash.harvard.edu/handle/1/37366886

5. Dagan N, Barda N, Kepten E, et al. BNT162b2 mRNA Covid-19 Vaccine in a Nationwide Mass Vaccination Setting. *New England Journal of Medicine*. 2021;0(0):null. doi:10.1056/NEJMoa2101765

6. Fine JP, Gray RJ. A Proportional Hazards Model for the Subdistribution of a Competing Risk. *Journal of the American Statistical Association*. 1999;94(446):496-509. doi:10.1080/01621459.1999.10474144

7. Polack FP, Thomas SJ, Kitchin N, et al. Safety and Efficacy of the BNT162b2 mRNA Covid-19 Vaccine. *N Engl J Med*. 2020;383(27):2603-2615. doi:10.1056/NEJMoa2034577

© 2021 Wu JTY et al. *JAMA Oncology.*

eFigure 1. Flow Diagram of Cohort Selection and Matching.

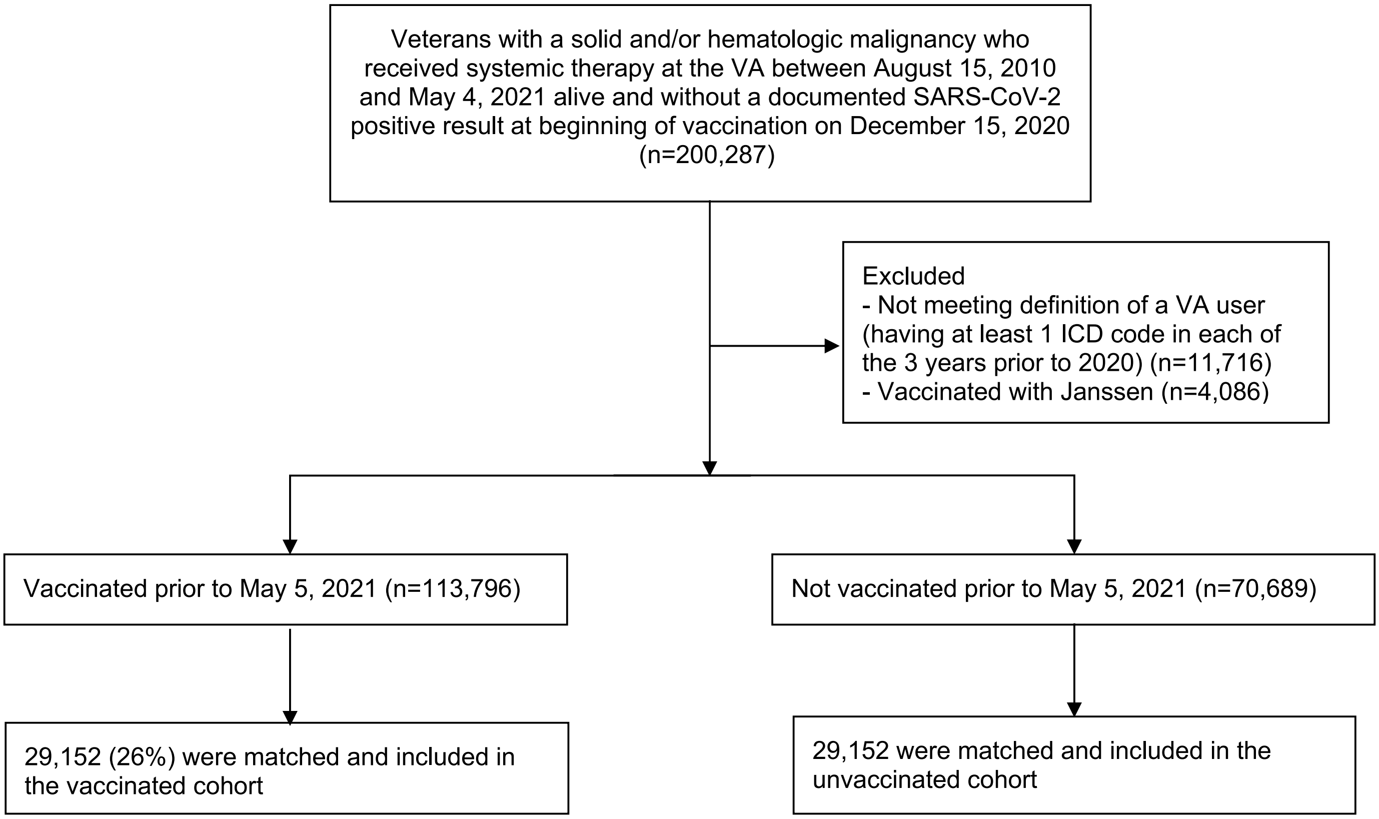

© 2021 Wu JTY et al. *JAMA Oncology.*

eFigure 2. Cumulative Incidence and Difference in SARS-COV-2 Infection between Matched Vaccinated and Unvaccinated Cohorts Stratified by Vaccination Type.

Cumulative incidence curves of SARS-COV-2 infection by vaccination type shown using time zero as the date of the first dose of vaccination. The difference in cumulative incidence is shown below. Confidence intervals were calculated by bootstrapping. The number at risk and cumulative number of events are also shown. A) Patients vaccinated with at least one dose of Pfizer BNT162b2 vaccine. B) Patients vaccinated with at least one dose of Moderna mRNA-1273 vaccine.

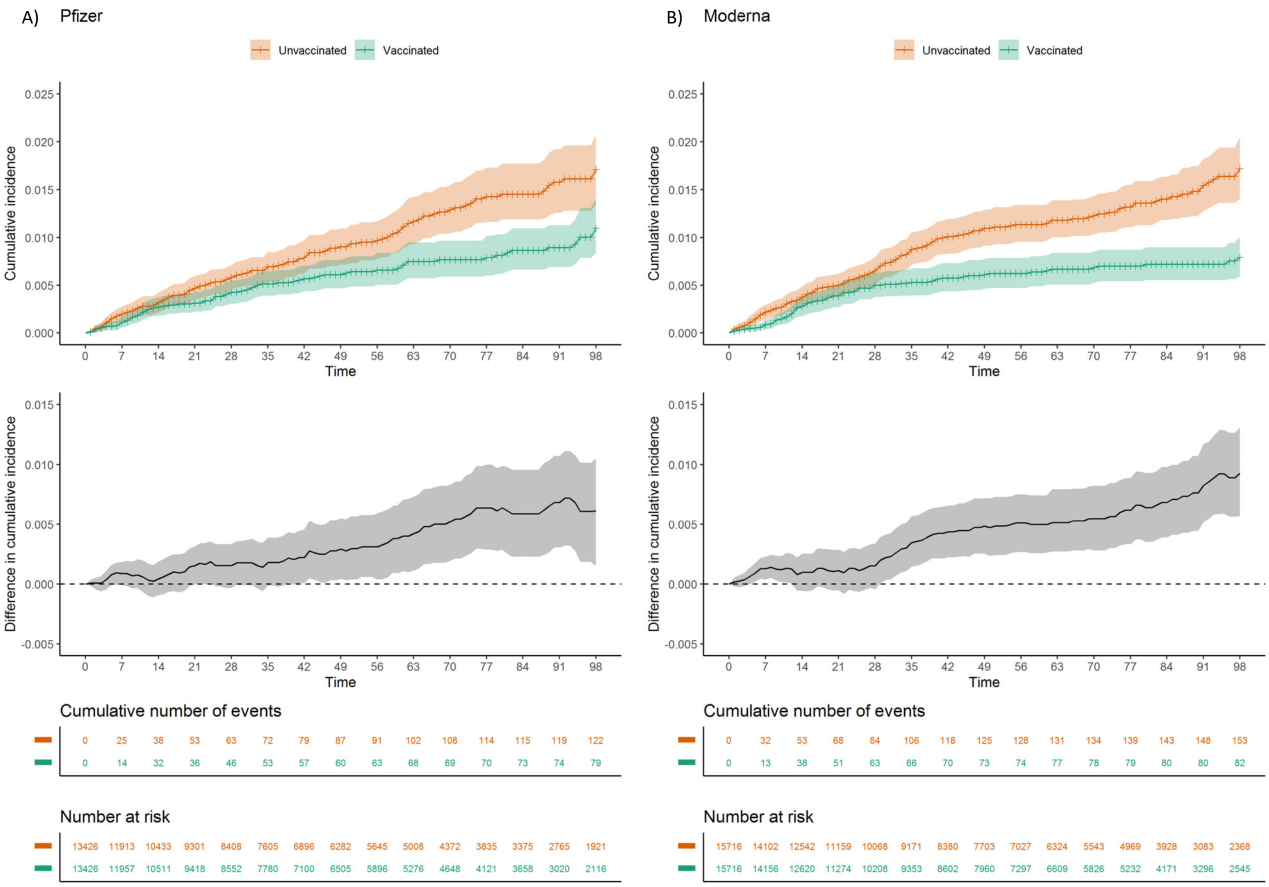

© 2021 Wu JTY et al. *JAMA Oncology.*

eFigure 3. Cumulative Incidence and Difference in SARS-COV-2 Infection between Matched Vaccinated and Unvaccinated Cohorts Stratified by Cancer Category.

Cumulative incidence curves of SARS-COV-2 infection by subgroups by broad cancer category defined in eMethods. Time zero is the date of the first dose of vaccination. The difference in cumulative incidence is shown below. Confidence intervals were calculated by bootstrapping. The number at risk and cumulative number of events are also shown. A) Solid malignancies as defined by cancer type in eMethods. B) Hematologic malignancies as defined by cancer type in eMethods.

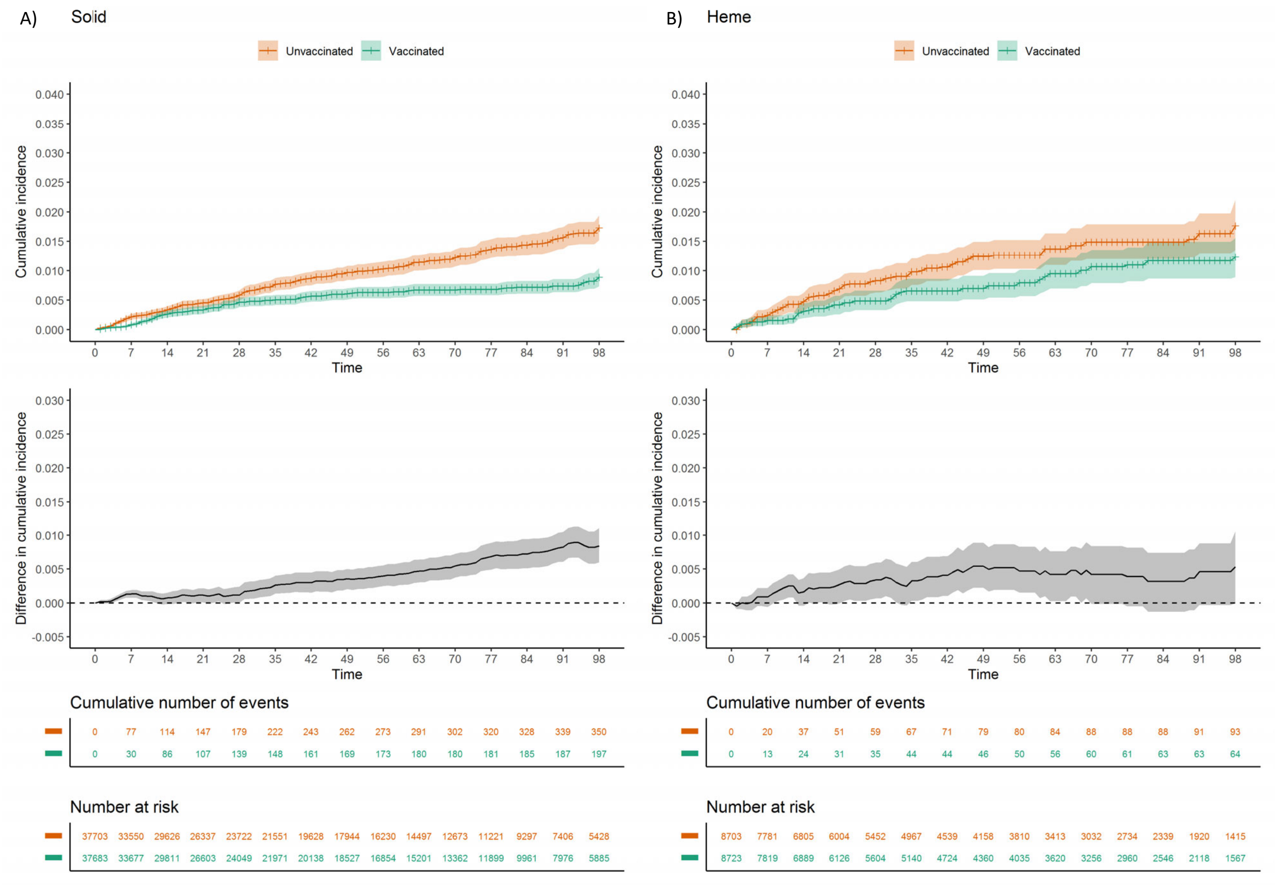

© 2021 Wu JTY et al. *JAMA Oncology.*

eFigure 4. Cumulative Incidence and Difference in SARS-COV-2 Infection between Matched Vaccinated and Unvaccinated Cohorts Stratified by Treatment Timing.

Cumulative incidence curves of SARS-COV-2 infection by subgroups by timing of therapy defined in eMethods. Time zero is the date of the first dose of vaccination. The difference in cumulative incidence is shown below. Confidence intervals were calculated by bootstrapping. The number at risk and cumulative number of events are also shown. A) Distant treatment, defined as systemic therapy received in the ten years prior to vaccination (if in the vaccinated cohort) or prior to entry date (if in the unvaccinated cohort) but not received in the six months prior to vaccination (if in the vaccinated cohort) or prior to entry date (if in the unvaccinated cohort). B) Recent treatment, defined as systemic therapy received in the three to six months prior to vaccination (if in the vaccinated cohort) or prior to entry date (if in the unvaccinated cohort) but not received in the three months prior to vaccination (if in the vaccinated cohort) or prior to entry date (if in the unvaccinated cohort). C) Current treatment, defined as systemic therapy received within the three months prior to vaccination (if in the vaccinated cohort) or prior to entry date (if in the unvaccinated cohort). D) Treatment received after vaccination, defined as first dose of systemic therapy received anytime after vaccination and matching and prior to study end date on May 4, 2021.

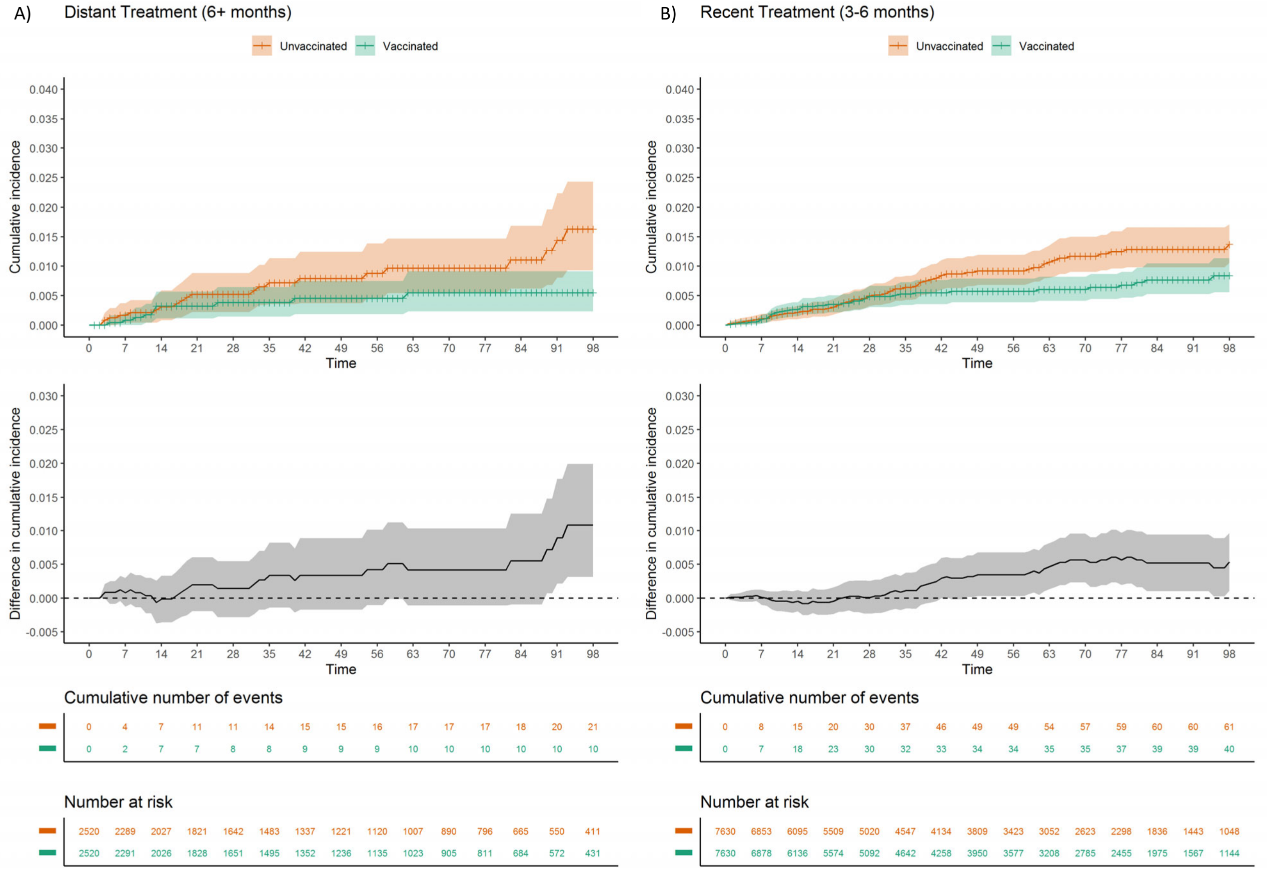

© 2021 Wu JTY et al. *JAMA Oncology.*

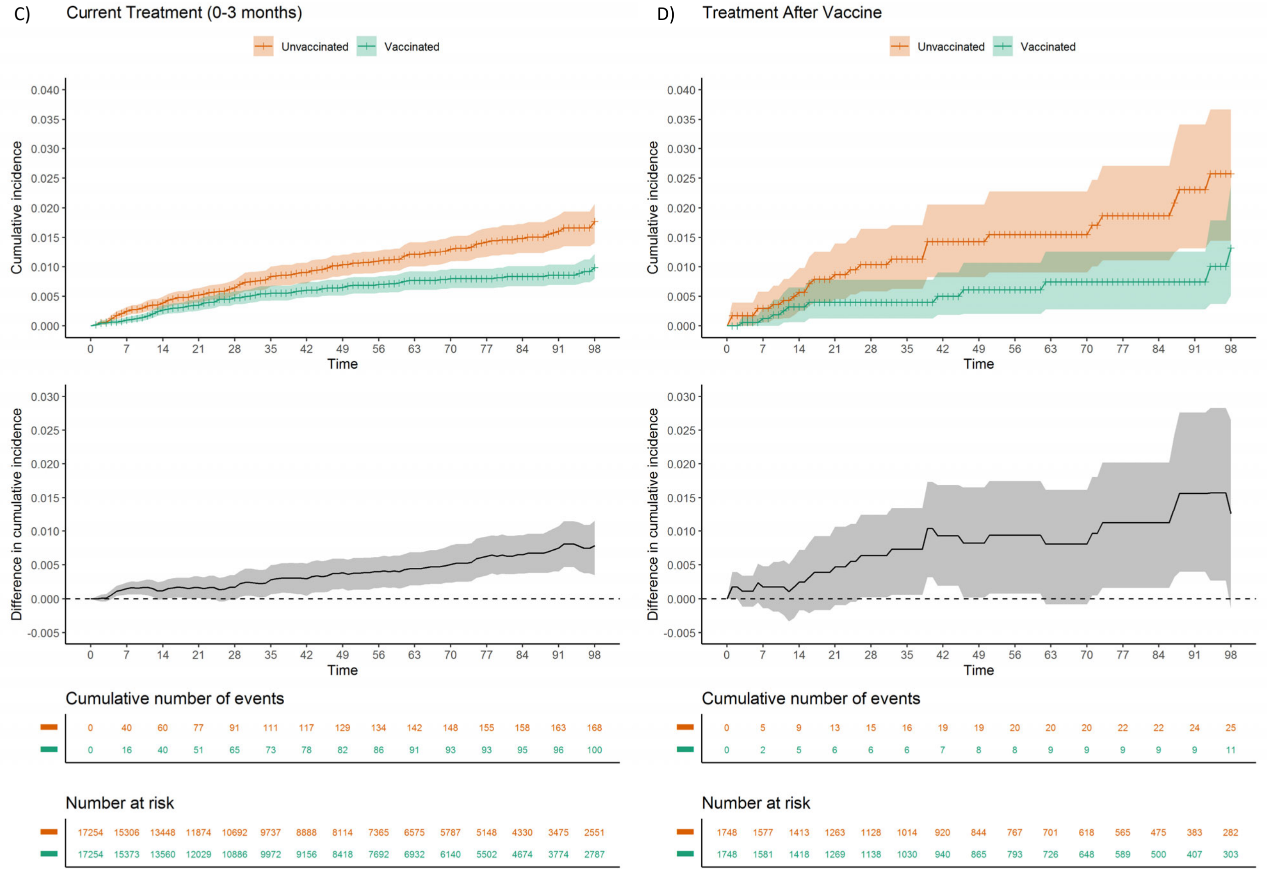

© 2021 Wu JTY et al. *JAMA Oncology.*

eFigure 5. Cumulative Incidence and Difference in SARS-COV-2 Infection between Matched Vaccinated and Unvaccinated Cohorts Stratified by Additional Treatment Types.

Cumulative incidence curves of SARS-COV-2 infection by current treatment type as defined in eMethods. shown using time zero as the date of the first dose of vaccination. The difference in cumulative incidence is shown below. Confidence intervals were calculated by bootstrapping. The number at risk and cumulative number of events are also shown. All patients in all panels on are on current treatment, defined as systemic therapy received within the three months prior to vaccination (if in the vaccinated cohort) or prior to entry date (if in the unvaccinated cohort). A) Patients on a targeted therapy at time of vaccination. B) Patients on an immunotherapy regimen.

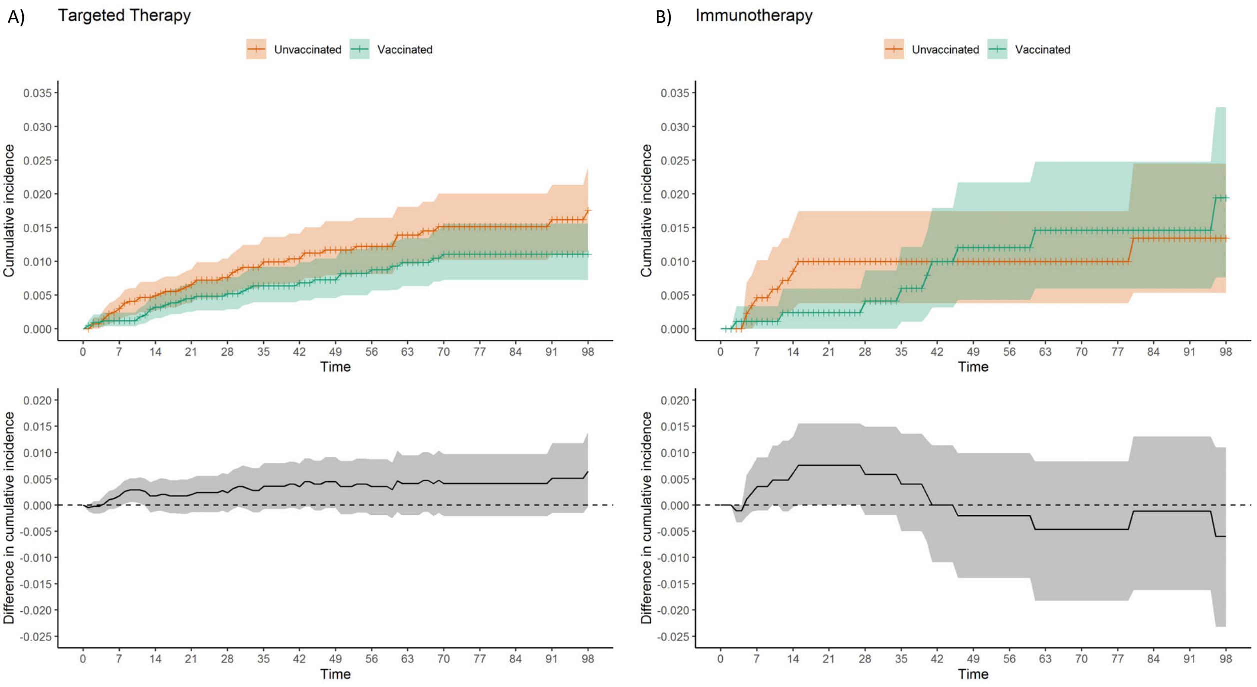

A) Targeted Therapy

Cumulative number of events

0	12	19	24	27	33	34	37	38	41	43	43	43	44	45
0	5	12	16	18	21	22	23	26	28	30	30	30	30	30

Number at risk

4292	3800	3334	2929	2635	2403	2189	1996	1816	1623	1430	1291	1106	913	678
4292	3813	3363	2968	2686	2466	2257	2077	1899	1703	1514	1374	1186	991	742

B) Immunotherapy

Cumulative number of events

0	4	7	8	8	8	8	8	8	8	8	8	9	9	9
0	1	2	2	3	4	6	7	7	8	8	8	8	8	9

Number at risk

979	834	706	608	540	487	445	398	354	312	283	253	221	185	139
979	848	727	635	572	521	479	433	392	353	321	292	263	221	163

© 2021 Wu JTY et al. *JAMA Oncology.*

eFigure 6. Cancer Type Determination Algorithm.

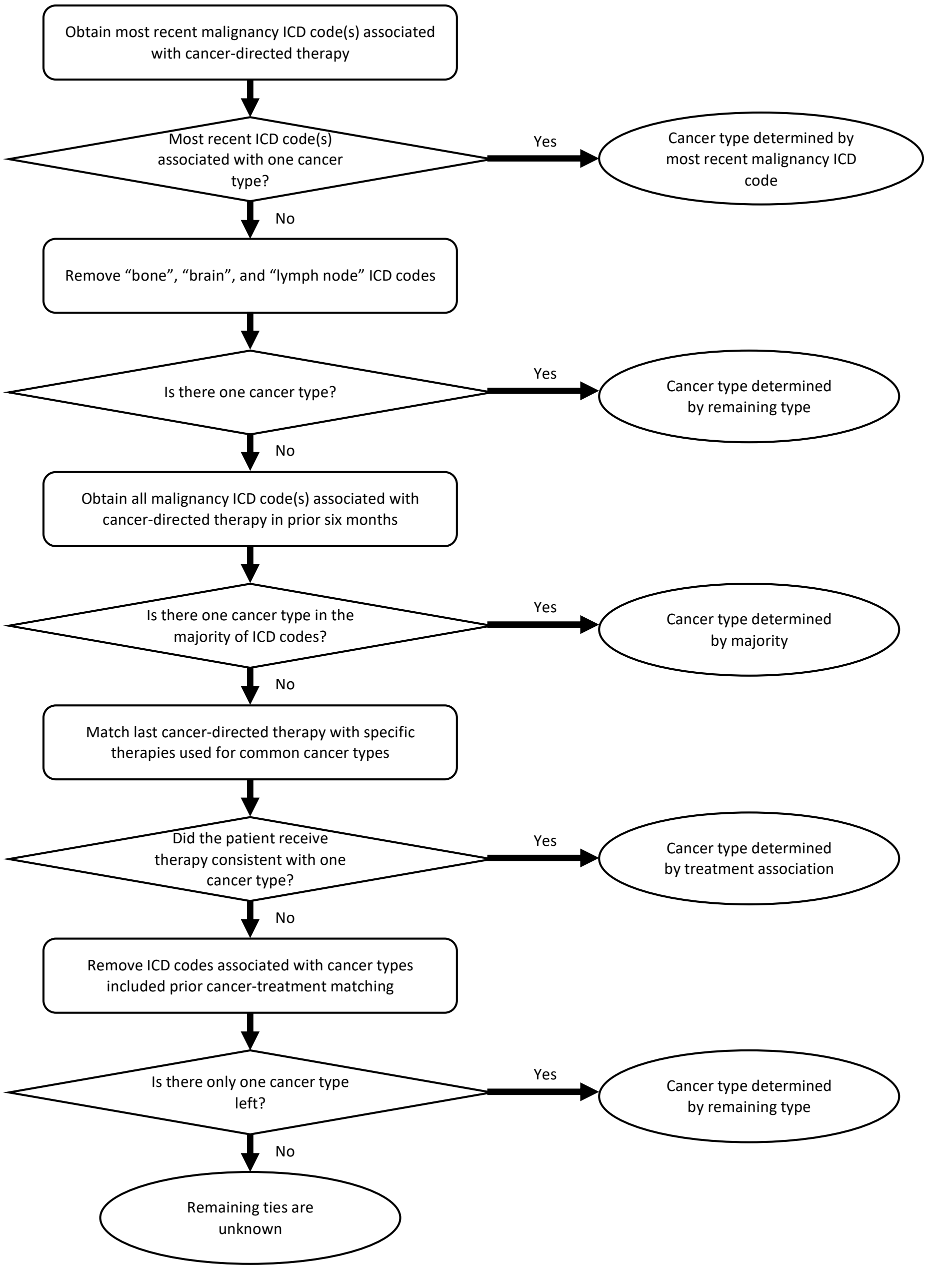

© 2021 Wu JTY et al. *JAMA Oncology.*

eTable 1. Demographic and Clinical Characteristics of Vaccinated and Unvaccinated VA Cancer Patients in the Pre-match Cohort[e]

Baseline Covariates	Overall (N=184485)	Unvaccinated (N=70689)	Vaccinated (N=113796)
Age (median [IQR])	73.13 [67.46, 78.12]	72.48 [65.35, 77.79]	73.44 [68.62, 78.26]
Age (%)			
<50	6626 (3.6)	4289 (6.1)	2337 (2.1)
50-59	12707 (6.9)	6348 (9.0)	6359 (5.6)
60-69	40595 (22.0)	15921 (22.5)	24674 (21.7)
70-79	87822 (47.6)	30115 (42.6)	57707 (50.7)
>=80	36735 (19.9)	14016 (19.8)	22719 (20.0)
Gender = M (%)	169645 (92.0)	63768 (90.2)	105877 (93.0)
Race/Ethnicity (%)			
Non-Hispanic White	126934 (68.8)	49371 (69.8)	77563 (68.2)
Hispanic	8811 (4.8)	2975 (4.2)	5836 (5.1)
Non-Hispanic Black	36671 (19.9)	13326 (18.9)	23345 (20.5)
Other or Unk	12069 (6.5)	5017 (7.1)	7052 (6.2)
Rurality[a] (%)			
Rural	62643 (34.0)	27900 (39.5)	34743 (30.5)
Urban	120163 (65.1)	42157 (59.6)	78006 (68.5)
Unknown	1679 (0.9)	632 (0.9)	1047 (0.9)
Region[b] (%)			
North Atlantic	40964 (22.2)	14120 (20.0)	26844 (23.6)
Southeast	42223 (22.9)	17258 (24.4)	24965 (21.9)
Midwest	40268 (21.8)	14350 (20.3)	25918 (22.8)
Continental	29023 (15.7)	13130 (18.6)	15893 (14.0)
Pacific	32007 (17.3)	11831 (16.7)	20176 (17.7)
Charlson Score (%)			
0-2	122936 (66.6)	50430 (71.3)	72506 (63.7)
3-4	44988 (24.4)	14910 (21.1)	30078 (26.4)
5+	16561 (9.0)	5349 (7.6)	11212 (9.9)
Vaccine Type[c] (%)			
Moderna	-	-	62376 (54.8)
Pfizer	-	-	51389 (45.2)
Unknown	-	-	31 (0.0)
Cancer type[d] (%)			
Prostate	66751 (36.2)	25198 (35.6)	41553 (36.5)
SCCHN/CSCC/skin	29937 (16.2)	11343 (16)	18594 (16.3)
Lung	10090 (5.5)	3418 (4.8)	6672 (5.9)
Leukemia/MDS/MF	9373 (5.1)	3727 (5.3)	5646 (5)
Colorectal	9307 (5)	3698 (5.2)	5609 (4.9)
Lymphoma	8676 (4.7)	3195 (4.5)	5481 (4.8)
Breast	8291 (4.5)	3598 (5.1)	4693 (4.1)
Multiple myeloma	6322 (3.4)	2241 (3.2)	4081 (3.6)
Urothelial	6269 (3.4)	2051 (2.9)	4218 (3.7)
HCC	3377 (1.8)	1218 (1.7)	2159 (1.9)
Esophagus/gastric	2807 (1.5)	993 (1.4)	1814 (1.6)

© 2021 Wu JTY et al. *JAMA Oncology.*

Gyn	2560 (1.4)	1547 (2.2)	1013 (0.9)
Melanoma	2496 (1.4)	950 (1.3)	1546 (1.4)
Essential thrombocythemia	2333 (1.3)	971 (1.4)	1362 (1.2)
Neuroendocrine	2019 (1.1)	754 (1.1)	1265 (1.1)
RCC	1966 (1.1)	748 (1.1)	1218 (1.1)
Polycythemia vera	1936 (1)	811 (1.1)	1125 (1)
Other GI	1453 (0.8)	559 (0.8)	894 (0.8)
Brain	834 (0.5)	430 (0.6)	404 (0.4)
Connective and soft tissue	419 (0.2)	181 (0.3)	238 (0.2)
Other/unknown/lesser	7269 (3.9)	3058 (4.3)	4211 (3.7)

[a] Defined based on patient home address.
[b] Defined based on VA station at which patient received cancer care.
[c] Vaccination type only shown for overall and vaccinated cohort, even though patients in the unvaccinated cohort can be subsequently vaccinated after matching, as those vaccinations are already tabulated in the vaccinated cohort.
[d] Cancer type determined by ICD code(s) associated with cancer-directed systemic treatment as detailed in eMethods. Other/unknown/lesser includes cancer of unknown primary, cancer types not included in a predefined category, and cancer types represented by 50 patients or less. CSCC = cutaneous squamous cell carcinoma; Gyn = gynecologic malignancy; HCC = hepatocellular carcinoma; MF = myelofibrosis; MDS = myelodysplastic syndrome; Other GI = anal/biliary/GIST/pancreas/small intestine; RCC = renal cell carcinoma; SCCHN = squamous cell head and neck cancer.
[e] Systemic therapy not included in this table as it was a rolling variable defined in relation to vaccination/matching time.

© 2021 Wu JTY et al. *JAMA Oncology.*

eTable 2. Demographic and Clinical Characteristics of Vaccinated and Unvaccinated VA Cancer Patients in the Matched Cohort.

Baseline Covariates	Overall (N=58304)	Unvaccinated (N=29152)	Vaccinated (N=29152)
Age (median [IQR])	73.73 [69.43, 78.62]	73.31 [68.59, 77.87]	74.14 [70.21, 79.32]
Age (%)			
<50	943 (1.6)	612 (2.1)	331 (1.1)
50-59	2830 (4.9)	1652 (5.7)	1178 (4.0)
60-69	11828 (20.3)	6314 (21.7)	5514 (18.9)
70-79	30366 (52.1)	14978 (51.4)	15388 (52.8)
>=80	12337 (21.2)	5596 (19.2)	6741 (23.1)
Gender, Male (%)	55029 (94.4)	27408 (94.0)	27621 (94.7)
Race/ethnicity			
Non-Hispanic White	41402 (71.0)	20701 (71.0)	20701 (71.0)
Hispanic	2364 (4.1)	1182 (4.1)	1182 (4.1)
Non-Hispanic Black	12334 (21.2)	6167 (21.2)	6167 (21.2)
Other or Unk	2204 (3.8)	1102 (3.8)	1102 (3.8)
Rurality[a] (%)			
Rural	17740 (30.4)	8870 (30.4)	8870 (30.4)
Urban	40322 (69.2)	20161 (69.2)	20161 (69.2)
Unknown	242 (0.4)	121 (0.4)	121 (0.4)
Region[b] (%)			
North Atlantic	13234 (22.7)	6617 (22.7)	6617 (22.7)
Southeast	13146 (22.5)	6573 (22.5)	6573 (22.5)
Midwest	14414 (24.7)	7207 (24.7)	7207 (24.7)
Continental	7700 (13.2)	3850 (13.2)	3850 (13.2)
Pacific	9810 (16.8)	4905 (16.8)	4905 (16.8)
Charlson Score (%)			
0-2	34864 (59.8)	18063 (62.0)	16801 (57.6)
3-4	17082 (29.3)	8208 (28.2)	8874 (30.4)
5+	6358 (10.9)	2881 (9.9)	3477 (11.9)
Vaccine Type[c] (%)			
Moderna	-	-	15716 (53.9)
Pfizer	-	-	13426 (46.1)
Unknown	-	-	10 (0.0)
Systemic therapy received[d]			
Distant treatment (>6 Months)	5040 (8.6)	2520 (8.6)	2520 (8.6)
Recent treatment (3-6 Months)	15260 (26.2)	7630 (26.2)	7630 (26.2)
Current chemotherapy-Containing (<3 Months)	8452 (14.5)	4226 (14.5)	4226 (14.5)
Current immunotherapy (<3 Months)	1958 (3.4)	979 (3.4)	979 (3.4)
Current targeted (<3 Months)	8584 (14.7)	4292 (14.7)	4292 (14.7)

© 2021 Wu JTY et al. *JAMA Oncology.*

Current endocrine (<3 Months)	15514 (26.6)	7757 (26.6)	7757 (26.6)
Treatment after vaccine	3496 (6.0)	1748 (6.0)	1748 (6.0)
Cancer type[e] (%)			
Prostate	27848 (47.8)	13924 (47.8)	13924 (47.8)
SCCHN/CSCC/skin	5310 (9.1)	2655 (9.1)	2655 (9.1)
Leukemia/MDS/MF	4986 (8.6)	2493 (8.6)	2493 (8.6)
Lung	3692 (6.3)	1846 (6.3)	1846 (6.3)
Multiple myeloma	3212 (5.5)	1606 (5.5)	1606 (5.5)
Breast	2358 (4)	1179 (4)	1179 (4)
Urothelial	1756 (3)	878 (3)	878 (3)
Lymphoma	1504 (2.6)	752 (2.6)	752 (2.6)
Colorectal	1466 (2.5)	733 (2.5)	733 (2.5)
Essential thrombocythemia	904 (1.6)	452 (1.6)	452 (1.6)
HCC	746 (1.3)	373 (1.3)	373 (1.3)
Polycythemia vera	622 (1.1)	311 (1.1)	311 (1.1)
Esophagus/gastric	510 (0.9)	255 (0.9)	255 (0.9)
Melanoma	470 (0.8)	235 (0.8)	235 (0.8)
RCC	450 (0.8)	225 (0.8)	225 (0.8)
Neuroendocrine	420 (0.7)	210 (0.7)	210 (0.7)
Other GI	316 (0.5)	158 (0.5)	158 (0.5)
Gyn	102 (0.2)	51 (0.2)	51 (0.2)
Other/unknown/lesser	1632 (2.8)	816 (2.8)	816 (2.8)

[a] Defined based on patient home address.

[b] Defined based on VA station at which patient received cancer care.

[c] Vaccination type only shown for vaccinated cohort, even though patients in the unvaccinated cohort can be subsequently vaccinated after matching, as those vaccinations are already tabulated in the vaccinated cohort.

[d] Systemic therapy received relative to vaccination (if in the vaccinated cohort) or entry date (if in the unvaccinated cohort) with timing determined by the last dose of systemic therapy received. Current treatment, defined as systemic therapy received within three months prior to vaccination, was further subdivided into different treatment types as detailed in eMethods.

[e] Cancer type determined by ICD code(s) associated with cancer-directed systemic treatment as detailed in eMethods. Other/unknown/lesser includes cancer of unknown primary, cancer types not included in a predefined category, and cancer types represented by 50 patients or less. CSCC = cutaneous squamous cell carcinoma; Gyn = gynecologic malignancy; HCC = hepatocellular carcinoma; MF = myelofibrosis; MDS = myelodysplastic syndrome; Other GI = anal/biliary/GIST/pancreas/small intestine; SCCHN = squamous cell head and neck cancer; RCC = renal cell carcinoma.

© 2021 Wu JTY et al. *JAMA Oncology.*

eTable 3. Estimated Vaccine Effectiveness against SARS-COV-2 Infection with Alternate Time Periods.

Group and period	1-RR (95% CI)
**Overall**	
0 days after first dose to end of study	42 (28 to 53)
0 days after first dose to day of second dose	41 (24 to 56)
14 days after first dose to day of second dose	45 (8 to 66)
0 days to 13 days after second dose[a]	57 (28 to 76)
**Solid malignancy**	
0 days after first dose to end of study	44 (30 to 56)
0 days after first dose to day of second dose	38 (15 to 55)
14 days after first dose to day of second dose	41 (1 to 66)
0 days to 13 days after second dose[a]	58 (24 to 79)
**Hematologic malignancy**	
0 days after first dose to end of study	38 (5 to 59)
0 days after first dose to day of second dose	53 (16 to 77)
14 days after first dose to day of second dose	59 (-3 to 90)
0 days to 13 days after second dose[a]	57 (-38 to 92)
**Distant treatment**	
0 days after first dose to end of study	56 (13 to 82)
0 days after first dose to day of second dose	37 (-68 to 83)
14 days after first dose to day of second dose	-- [b]
0 days to 13 days after second dose[a]	-- [b]
**Recent treatment**	
0 days after first dose to end of study	35 (5 to 56)
0 days after first dose to day of second dose	17 (-50 to 54)
14 days after first dose to day of second dose	37 (-51 to 77)
0 days to 13 days after second dose[a]	57 (-6 to 86)
**Current treatment**	
0 days after first dose to end of study	41 (23 to 55)
0 days after first dose to day of second dose	46 (22 to 64)
14 days after first dose to day of second dose	29 (-29 to 62)
0 days to 13 days after second dose[a]	54 (12 to 80)
**Treatment after vaccine**	
0 days after first dose to end of study	56 (13 to 80)
0 days after first dose to day of second dose	66 (15 to 93)
14 days after first dose to day of second dose	-- [b]
0 days to 13 days after second dose[a]	-- [b]
**Current chemotherapy-containing**	
0 days after first dose to end of study	24 (-27 to 55)
0 days after first dose to day of second dose	24 (-60 to 69)
14 days after first dose to day of second dose	-71 (-830 to 43)
0 days to 13 days after second dose[a]	-- [b]
**Current targeted**	
0 days after first dose to end of study	31 (-9 to 58)
0 days after first dose to day of second dose	50 (-1 to 79)
14 days after first dose to day of second dose	-- [b]

© 2021 Wu JTY et al. *JAMA Oncology.*

0 days to 13 days after second dose[a]	61 (-45 to 100)
**Current endocrine**	
0 days after first dose to end of study	57 (38 to 73)
0 days after first dose to day of second dose	46 (1 to 72)
14 days after first dose to day of second dose	64 (8 to 93)
0 days to 13 days after second dose[a]	72 (23 to 95)

[a] Patients who received a second dose of vaccination prior to 14 days after the first dose excluded.
[b] Not enough SARS-COV-2 infection events to estimate effectiveness.

© 2021 Wu JTY et al. *JAMA Oncology.*

eTable 4. Demographic and Clinical Characteristics of Matched Vaccinated and Unvaccinated VA Cancer Patients with Solid Malignancies.

Baseline Covariates	Overall (N=48185)	Unvaccinated[a] (N=24103)	Vaccinated (N=24082)
Age (median [IQR])	73.78 [69.50, 78.84]	73.37 [68.72, 78.17]	74.18 [70.28, 79.54]
Age (%)			
<50	727 (1.5)	469 (1.9)	258 (1.1)
50-59	2220 (4.6)	1287 (5.3)	933 (3.9)
60-69	9799 (20.3)	5252 (21.8)	4547 (18.9)
70-79	24901 (51.7)	12286 (51.0)	12615 (52.4)
>=80	10538 (21.9)	4809 (20.0)	5729 (23.8)
Gender = M (%)	45246 (93.9)	22555 (93.6)	22691 (94.2)
Race/Ethnicity (%)			
Non-Hispanic White	33728 (70.0)	16877 (70.0)	16851 (70.0)
Hispanic	2035 (4.2)	1016 (4.2)	1019 (4.2)
Non-Hispanic Black	10530 (21.9)	5265 (21.8)	5265 (21.9)
Other or Unk	1892 (3.9)	945 (3.9)	947 (3.9)
Rurality[b] (%)			
Rural	14525 (30.1)	7269 (30.2)	7256 (30.1)
Urban	33434 (69.4)	16721 (69.4)	16713 (69.4)
Unknown	226 (0.5)	113 (0.5)	113 (0.5)
Region[c] (%)			
North Atlantic	10896 (22.6)	5453 (22.6)	5443 (22.6)
Southeast	11124 (23.1)	5563 (23.1)	5561 (23.1)
Midwest	11772 (24.4)	5891 (24.4)	5881 (24.4)
Continental	6247 (13.0)	3124 (13.0)	3123 (13.0)
Pacific	8146 (16.9)	4072 (16.9)	4074 (16.9)
Charlson Score (%)			
0-2	28155 (58.4)	14591 (60.5)	13564 (56.3)
3-4	14605 (30.3)	7029 (29.2)	7576 (31.5)
5+	5425 (11.3)	2483 (10.3)	2942 (12.2)
Vaccine Type[d] (%)			
Moderna	-	-	12974 (53.9)
Pfizer	-	-	11101 (46.1)
Unknown	-	-	7 (0.0)
Systemic therapy received[e]			
Distant treatment (>6 Months)	4458 (9.3)	2234 (9.3)	2224 (9.2)
Recent treatment (3-6 Months)	13345 (27.7)	6676 (27.7)	6669 (27.7)
Current chemotherapy-Containing (<3 Months)	6875 (14.3)	3439 (14.3)	3436 (14.3)
Current immunotherapy (<3 Months)	1958 (4.1)	979 (4.1)	979 (4.1)
Current targeted (<3 Months)	2863 (5.9)	1430 (5.9)	1433 (6.0)
Current endocrine (<3 Months)	15505 (32.2)	7752 (32.2)	7753 (32.2)

© 2021 Wu JTY et al. *JAMA Oncology.*

Treatment after vaccine	3181 (6.6)	1593 (6.6)	1588 (6.6)
Cancer type[f] (%)			
Prostate	27848 (57.8)	13924 (57.8)	13924 (57.8)
SCCHN/CSCC/skin	5310 (11)	2655 (11)	2655 (11)
Lung	3692 (7.7)	1846 (7.7)	1846 (7.7)
Breast	2358 (4.9)	1179 (4.9)	1179 (4.9)
Urothelial	1756 (3.6)	878 (3.6)	878 (3.6)
Lymphoma	1504 (3.1)	752 (3.1)	752 (3.1)
Colorectal	1466 (3)	733 (3)	733 (3)
HCC	746 (1.5)	373 (1.5)	373 (1.5)
Esophagus/gastric	510 (1.1)	255 (1.1)	255 (1.1)
Melanoma	470 (1.0)	235 (1.0)	235 (1.0)
RCC	450 (0.9)	225 (0.9)	225 (0.9)
Neuroendocrine	420 (0.9)	210 (0.9)	210 (0.9)
Other GI	316 (0.7)	158 (0.7)	158 (0.7)
Gyn	102 (0.2)	51 (0.2)	51 (0.2)
Other/unknown/lesser	1237 (2.6)	629 (2.6)	608 (2.5)

[a] The number of vaccinated patients and unvaccinated patients do not match exactly because while "other/unknown" was used as one cancer type for matching, "other/unknown" was split into solid and hematologic malignancies by ICD code for the cancer category subanalysis.
[b] Defined based on patient home address.
[c] Defined based on VA station at which patient received cancer care.
[d] Vaccination type only shown for overall and vaccinated cohort, even though patients in the unvaccinated cohort can be subsequently vaccinated after matching, as those vaccinations are already tabulated in the vaccinated cohort.
[e] Systemic therapy received prior to vaccination (if in the vaccinated cohort) or prior to entry date (if in the unvaccinated cohort) with timing determined by the last dose of systemic therapy received. Current treatment, defined as systemic therapy received within three months prior to vaccination, was further subdivided into different treatment types as detailed in eMethods.
[f] Cancer type determined by ICD code(s) associated with cancer-directed systemic treatment as detailed in eMethods. Other/unknown/lesser includes cancer of unknown primary, cancer types not included in a predefined category, and cancer types represented by 50 patients or less. CSCC = cutaneous squamous cell carcinoma; Gyn = gynecologic malignancy; HCC = hepatocellular carcinoma; Other GI = anal/biliary/GIST/pancreas/small intestine; RCC = renal cell carcinoma; SCCHN = squamous cell head and neck cancer.

© 2021 Wu JTY et al. *JAMA Oncology.*

eTable 5. Demographic and Clinical Characteristics of Matched Vaccinated and Unvaccinated VA Cancer Patients with Hematologic Malignancies.

Baseline Covariates	Overall (N=10119)	Unvaccinated[a] (N=5049)	Vaccinated (N=5070)
Age (median [IQR])	73.46 [69.02, 77.62]	73.01 [67.84, 76.83]	73.92 [69.90, 78.39]
Age (%)			
<50	216 (2.1)	143 (2.8)	73 (1.4)
50-59	610 (6.0)	365 (7.2)	245 (4.8)
60-69	2029 (20.1)	1062 (21.0)	967 (19.1)
70-79	5465 (54.0)	2692 (53.3)	2773 (54.7)
>=80	1799 (17.8)	787 (15.6)	1012 (20.0)
Gender = M (%)	9783 (96.7)	4853 (96.1)	4930 (97.2)
Race/Ethnicity (%)			
Non-Hispanic White	7674 (75.8)	3824 (75.7)	3850 (75.9)
Hispanic	329 (3.3)	166 (3.3)	163 (3.2)
Non-Hispanic Black	1804 (17.8)	902 (17.9)	902 (17.8)
Other or Unk	312 (3.1)	157 (3.1)	155 (3.1)
Rurality[b] (%)			
Rural	3215 (31.8)	1601 (31.7)	1614 (31.8)
Urban	6888 (68.1)	3440 (68.1)	3448 (68.0)
Unknown	16 (0.2)	8 (0.2)	8 (0.2)
Region[c] (%)			
North Atlantic	2338 (23.1)	1164 (23.1)	1174 (23.2)
Southeast	2022 (20.0)	1010 (20.0)	1012 (20.0)
Midwest	2642 (26.1)	1316 (26.1)	1326 (26.2)
Continental	1453 (14.4)	726 (14.4)	727 (14.3)
Pacific	1664 (16.4)	833 (16.5)	831 (16.4)
Charlson Score (%)			
0-2	6709 (66.3)	3472 (68.8)	3237 (63.8)
3-4	2477 (24.5)	1179 (23.4)	1298 (25.6)
5+	933 (9.2)	398 (7.9)	535 (10.6)
Vaccine Type[d] (%)			
Moderna	-	-	2742 (54.1)
Pfizer	-	-	2325 (45.9)
Unknown	-	-	3 (0.1)
Systemic therapy received[e]			
Distant treatment (>6 Months)	582 (5.8)	286 (5.7)	296 (5.8)
Recent treatment (3-6 Months)	1915 (18.9)	954 (18.9)	961 (19.0)
Current chemotherapy-Containing (<3 Months)	1577 (15.6)	787 (15.6)	790 (15.6)
Current targeted (<3 Months)	5721 (56.5)	2862 (56.7)	2859 (56.4)
Current endocrine (<3 Months)	9 (0.1)	5 (0.1)	4 (0.1)
Treatment after vaccine	315 (3.1)	155 (3.1)	160 (3.2)
Cancer type[f] (%)			

© 2021 Wu JTY et al. *JAMA Oncology.*

Leukemia/MDS/MF	4986 (49.3)	2493 (49.4)	2493 (49.2)
Multiple myeloma	3212 (31.7)	1606 (31.8)	1606 (31.7)
Essential thrombocythemia	904 (8.9)	452 (9)	452 (8.9)
Polycythemia vera	622 (6.1)	311 (6.2)	311 (6.1)
Other/unknown/lesser	395 (3.9)	187 (3.7)	208 (4.1)

[a] The number of vaccinated patients and unvaccinated patients do not match exactly because while “other/unknown” was used as one cancer type for matching, “other/unknown” was split into solid and hematologic malignancies by ICD code for the cancer category subanalysis.
[b] Defined based on patient home address.
[c] Defined based on VA station at which patient received cancer care.
[d] Vaccination type only shown for overall and vaccinated cohort, even though patients in the unvaccinated cohort can be subsequently vaccinated after matching, as those vaccinations are already tabulated in the vaccinated cohort.
[e] Systemic therapy received prior to vaccination (if in the vaccinated cohort) or prior to entry date (if in the unvaccinated cohort) with timing determined by the last dose of systemic therapy received. Current treatment, defined as systemic therapy received within three months prior to vaccination, was further subdivided into different treatment types as detailed in eMethods.
[f] Cancer type determined by ICD code(s) associated with cancer-directed systemic treatment as detailed in eMethods. Other/unknown/lesser includes cancer of unknown primary, cancer types not included in a predefined category, and cancer types represented by 50 patients or less. MF = myelofibrosis; MDS = myelodysplastic syndrome.

© 2021 Wu JTY et al. *JAMA Oncology.*

eTable 6. Demographic and Clinical Characteristics of Matched Vaccinated and Unvaccinated VA Cancer Patients with Distant Treatment (>6 months).

Baseline Covariates	Overall (N=5040)	Unvaccinated (N=2520)	Vaccinated (N=2520)
Age (median [IQR])	74.35 [70.05, 80.53]	73.84 [69.06, 79.58]	75.25 [70.94, 81.36]
Age (%)			
<50	85 (1.7)	58 (2.3)	27 (1.1)
50-59	236 (4.7)	132 (5.2)	104 (4.1)
60-69	926 (18.4)	510 (20.2)	416 (16.5)
70-79	2452 (48.7)	1218 (48.3)	1234 (49.0)
>=80	1341 (26.6)	602 (23.9)	739 (29.3)
Gender = M (%)	4665 (92.6)	2330 (92.5)	2335 (92.7)
Race/Ethnicity (%)			
Non-Hispanic White	3782 (75.0)	1891 (75.0)	1891 (75.0)
Hispanic	176 (3.5)	88 (3.5)	88 (3.5)
Non-Hispanic Black	958 (19.0)	479 (19.0)	479 (19.0)
Other or Unk	124 (2.5)	62 (2.5)	62 (2.5)
Rurality[a] (%)			
Rural	1536 (30.5)	768 (30.5)	768 (30.5)
Urban	3482 (69.1)	1741 (69.1)	1741 (69.1)
Unknown	22 (0.4)	11 (0.4)	11 (0.4)
Region[b] (%)			
North Atlantic	1058 (21.0)	529 (21.0)	529 (21.0)
Southeast	1214 (24.1)	607 (24.1)	607 (24.1)
Midwest	1162 (23.1)	581 (23.1)	581 (23.1)
Continental	672 (13.3)	336 (13.3)	336 (13.3)
Pacific	934 (18.5)	467 (18.5)	467 (18.5)
Charlson Score (%)			
0-2	3350 (66.5)	1721 (68.3)	1629 (64.6)
3-4	1244 (24.7)	602 (23.9)	642 (25.5)
5+	446 (8.8)	197 (7.8)	249 (9.9)
Vaccine Type[c] (%)			
Moderna	-	-	1388 (55.1)
Pfizer	-	-	1132 (44.9)
Unknown	-	-	0 (0.0)
Cancer type[d] (%)			
Prostate	2716 (53.9)	1358 (53.9)	1358 (53.9)
SCCHN/CSCC/skin	640 (12.7)	320 (12.7)	320 (12.7)
Leukemia/MDS/MF	336 (6.7)	168 (6.7)	168 (6.7)
Breast	298 (5.9)	149 (5.9)	149 (5.9)
Lung	196 (3.9)	98 (3.9)	98 (3.9)
Urothelial	160 (3.2)	80 (3.2)	80 (3.2)
Lymphoma	102 (2.0)	51 (2.0)	51 (2.0)
Multiple myeloma	88 (1.7)	44 (1.7)	44 (1.7)
Colorectal	78 (1.5)	39 (1.5)	39 (1.5)
Essential thrombocythemia	66 (1.3)	33 (1.3)	33 (1.3)
Polycythemia vera	52 (1.0)	26 (1.0)	26 (1.0)

© 2021 Wu JTY et al. *JAMA Oncology.*

Other/unknown/lesser	308 (6.1)	154 (6.1)	154 (6.1)

[a] Defined based on patient home address.
[b] Defined based on VA station at which patient received cancer care.
[c] Vaccination type only shown for overall and vaccinated cohort, even though patients in the unvaccinated cohort can be subsequently vaccinated after matching, as those vaccinations are already tabulated in the vaccinated cohort.
[d] Cancer type determined by ICD code(s) associated with cancer-directed systemic treatment as detailed in eMethods. Other/unknown/lesser includes cancer of unknown primary, cancer types not included in a predefined category, and cancer types represented by 50 patients or less. CSCC = cutaneous squamous cell carcinoma; MF = myelofibrosis; MDS = myelodysplastic syndrome; SCCHN = squamous cell head and neck cancer.

© 2021 Wu JTY et al. *JAMA Oncology.*

eTable 7. Demographic and Clinical Characteristics of Matched Vaccinated and Unvaccinated VA Cancer Patients with Recent Treatment (3-6 Months).

Baseline Covariates	Overall (N=15260)	Unvaccinated (N=7630)	Vaccinated (N=7630)
Age (median [IQR])	73.67 [69.40, 78.67]	73.25 [68.60, 78.10]	74.03 [70.14, 79.30]
Age (%)			
<50	269 (1.8)	177 (2.3)	92 (1.2)
50-59	715 (4.7)	425 (5.6)	290 (3.8)
60-69	3102 (20.3)	1615 (21.2)	1487 (19.5)
70-79	7867 (51.6)	3878 (50.8)	3989 (52.3)
>=80	3307 (21.7)	1535 (20.1)	1772 (23.2)
Gender = M (%)	14235 (93.3)	7091 (92.9)	7144 (93.6)
Race/Ethnicity (%)			
Non-Hispanic White	10678 (70.0)	5339 (70.0)	5339 (70.0)
Hispanic	624 (4.1)	312 (4.1)	312 (4.1)
Non-Hispanic Black	3362 (22.0)	1681 (22.0)	1681 (22.0)
Other or Unk	596 (3.9)	298 (3.9)	298 (3.9)
Rurality[a] (%)			
Rural	4966 (32.5)	2483 (32.5)	2483 (32.5)
Urban	10238 (67.1)	5119 (67.1)	5119 (67.1)
Unknown	56 (0.4)	28 (0.4)	28 (0.4)
Region[b] (%)			
North Atlantic	3388 (22.2)	1694 (22.2)	1694 (22.2)
Southeast	3610 (23.7)	1805 (23.7)	1805 (23.7)
Midwest	3662 (24.0)	1831 (24.0)	1831 (24.0)
Continental	2114 (13.9)	1057 (13.9)	1057 (13.9)
Pacific	2486 (16.3)	1243 (16.3)	1243 (16.3)
Charlson Score (%)			
0-2	9418 (61.7)	4861 (63.7)	4557 (59.7)
3-4	4324 (28.3)	2065 (27.1)	2259 (29.6)
5+	1518 (9.9)	704 (9.2)	814 (10.7)
Vaccine Type[c] (%)			
Moderna	-	-	4207 (55.1)
Pfizer	-	-	3421 (44.8)
Unknown	-	-	2 (0.0)
Cancer type[d] (%)			
Prostate	8372 (54.9)	4186 (54.9)	4186 (54.9)
SCCHN/CSCC/skin	1638 (10.7)	819 (10.7)	819 (10.7)
Leukemia/MDS/MF	1106 (7.2)	553 (7.2)	553 (7.2)
Breast	798 (5.2)	399 (5.2)	399 (5.2)
Urothelial	492 (3.2)	246 (3.2)	246 (3.2)
Lung	460 (3.0)	230 (3.0)	230 (3.0)
Colorectal	324 (2.1)	162 (2.1)	162 (2.1)
Lymphoma	300 (2.0)	150 (2.0)	150 (2.0)
Essential thrombocythemia	294 (1.9)	147 (1.9)	147 (1.9)
Multiple myeloma	210 (1.4)	105 (1.4)	105 (1.4)
Polycythemia vera	182 (1.2)	91 (1.2)	91 (1.2)

© 2021 Wu JTY et al. *JAMA Oncology.*

HCC	180 (1.2)	90 (1.2)	90 (1.2)
Neuroendocrine	98 (0.6)	49 (0.6)	49 (0.6)
Melanoma	96 (0.6)	48 (0.6)	48 (0.6)
Esophagus/gastric	88 (0.6)	44 (0.6)	44 (0.6)
RCC	70 (0.5)	35 (0.5)	35 (0.5)
Other/unknown/lesser	552 (3.6)	276 (3.6)	276 (3.6)

[a] Defined based on patient home address.
[b] Defined based on VA station at which patient received cancer care.
[c] Vaccination type only shown for overall and vaccinated cohort, even though patients in the unvaccinated cohort can be subsequently vaccinated after matching, as those vaccinations are already tabulated in the vaccinated cohort.
[d] Cancer type determined by ICD code(s) associated with cancer-directed systemic treatment as detailed in eMethods. Other/unknown/lesser includes cancer of unknown primary, cancer types not included in a predefined category, and cancer types represented by 50 patients or less. CSCC = cutaneous squamous cell carcinoma; HCC = hepatocellular carcinoma; MF = myelofibrosis; MDS = myelodysplastic syndrome; RCC = renal cell carcinoma; SCCHN = squamous cell head and neck cancer.

© 2021 Wu JTY et al. *JAMA Oncology.*

eTable 8. Demographic and Clinical Characteristics of Matched Vaccinated and Unvaccinated VA Cancer Patients with Current Treatment (<3 Months).

Baseline Covariates	Overall (N=34508)	Unvaccinated (N=17254)	Vaccinated (N=17254)
Age (median [IQR])	73.68 [69.37, 78.43]	73.32 [68.59, 77.76]	74.08 [70.13, 79.06]
Age (%)			
<50	548 (1.6)	346 (2.0)	202 (1.2)
50-59	1728 (5.0)	995 (5.8)	733 (4.2)
60-69	7045 (20.4)	3766 (21.8)	3279 (19.0)
70-79	18159 (52.6)	8935 (51.8)	9224 (53.5)
>=80	7028 (20.4)	3212 (18.6)	3816 (22.1)
Gender = M (%)	32731 (94.9)	16293 (94.4)	16438 (95.3)
Race/Ethnicity (%)			
Non-Hispanic White	24204 (70.1)	12102 (70.1)	12102 (70.1)
Hispanic	1460 (4.2)	730 (4.2)	730 (4.2)
Non-Hispanic Black	7466 (21.6)	3733 (21.6)	3733 (21.6)
Other or Unk	1378 (4.0)	689 (4.0)	689 (4.0)
Rurality[a] (%)			
Rural	10226 (29.6)	5113 (29.6)	5113 (29.6)
Urban	24128 (69.9)	12064 (69.9)	12064 (69.9)
Unknown	154 (0.4)	77 (0.4)	77 (0.4)
Region[b] (%)			
North Atlantic	7996 (23.2)	3998 (23.2)	3998 (23.2)
Southeast	7546 (21.9)	3773 (21.9)	3773 (21.9)
Midwest	8666 (25.1)	4333 (25.1)	4333 (25.1)
Continental	4464 (12.9)	2232 (12.9)	2232 (12.9)
Pacific	5836 (16.9)	2918 (16.9)	2918 (16.9)
Charlson Score (%)			
0-2	19573 (56.7)	10182 (59.0)	9391 (54.4)
3-4	10769 (31.2)	5199 (30.1)	5570 (32.3)
5+	4166 (12.1)	1873 (10.9)	2293 (13.3)
Vaccine Type[c] (%)			
Moderna	-	-	9214 (53.4)
Pfizer	-	-	8032 (46.6)
Unknown	-	-	8 (0.0)
Systemic therapy received[d]			
Current chemotherapy-Containing (<3 Months)	8452 (24.5)	4226 (24.5)	4226 (24.5)
Current immunotherapy (<3 Months)	1958 (5.7)	979 (5.7)	979 (5.7)
Current targeted (<3 Months)	8584 (24.9)	4292 (24.9)	4292 (24.9)
Current endocrine (<3 Months)	15514 (45.0)	7757 (45.0)	7757 (45.0)
Cancer type[e] (%)			
Prostate	15402 (44.6)	7701 (44.6)	7701 (44.6)
Leukemia/MDS/MF	3354 (9.7)	1677 (9.7)	1677 (9.7)
Multiple myeloma	2818 (8.2)	1409 (8.2)	1409 (8.2)

© 2021 Wu JTY et al. *JAMA Oncology.*

Lung	2538 (7.4)	1269 (7.4)	1269 (7.4)
SCCHN/CSCC/skin	2518 (7.3)	1259 (7.3)	1259 (7.3)
Breast	1228 (3.6)	614 (3.6)	614 (3.6)
Colorectal	992 (2.9)	496 (2.9)	496 (2.9)
Lymphoma	970 (2.8)	485 (2.8)	485 (2.8)
Urothelial	896 (2.6)	448 (2.6)	448 (2.6)
Essential thrombocythemia	532 (1.5)	266 (1.5)	266 (1.5)
HCC	408 (1.2)	204 (1.2)	204 (1.2)
Polycythemia vera	382 (1.1)	191 (1.1)	191 (1.1)
RCC	344 (1.0)	172 (1.0)	172 (1.0)
Esophagus/gastric	342 (1.0)	171 (1.0)	171 (1.0)
Melanoma	318 (0.9)	159 (0.9)	159 (0.9)
Neuroendocrine	266 (0.8)	133 (0.8)	133 (0.8)
Other GI	236 (0.7)	118 (0.7)	118 (0.7)
Gyn	54 (0.2)	27 (0.2)	27 (0.2)
Other/unknown/lesser	910 (2.6)	455 (2.6)	455 (2.6)

[a] Defined based on patient home address.
[b] Defined based on VA station at which patient received cancer care.
[c] Vaccination type only shown for overall and vaccinated cohort, even though patients in the unvaccinated cohort can be subsequently vaccinated after matching, as those vaccinations are already tabulated in the vaccinated cohort.
[d] Systemic therapy received prior to vaccination (if in the vaccinated cohort) or prior to entry date (if in the unvaccinated cohort) with timing determined by the last dose of systemic therapy received. Different treatment types defined as detailed in eMethods.
[e] Cancer type determined by ICD code(s) associated with cancer-directed systemic treatment as detailed in eMethods. Other/unknown/lesser includes cancer of unknown primary, cancer types not included in a predefined category, and cancer types represented by 50 patients or less. CSCC = cutaneous squamous cell carcinoma; Gyn = gynecologic malignancy; HCC = hepatocellular carcinoma; MF = myelofibrosis; MDS = myelodysplastic syndrome; Other GI = anal/biliary/GIST/pancreas/small intestine; RCC = renal cell carcinoma; SCCHN = squamous cell head and neck cancer.

© 2021 Wu JTY et al. *JAMA Oncology.*

eTable 9. Demographic and Clinical Characteristics of Matched Vaccinated and Unvaccinated VA Cancer Patients with Treatment After Vaccination.

Baseline Covariates	Overall (N=3496)	Unvaccinated (N=1748)	Vaccinated (N=1748)
Age (median [IQR])	73.55 [69.41, 77.87]	72.71 [68.00, 76.26]	74.33 [70.58, 79.42]
Age (%)			
<50	41 (1.2)	31 (1.8)	10 (0.6)
50-59	151 (4.3)	100 (5.7)	51 (2.9)
60-69	755 (21.6)	423 (24.2)	332 (19.0)
70-79	1888 (54.0)	947 (54.2)	941 (53.8)
>=80	661 (18.9)	247 (14.1)	414 (23.7)
Gender = M (%)	3398 (97.2)	1694 (96.9)	1704 (97.5)
Race/Ethnicity (%)			
Non-Hispanic White	2738 (78.3)	1369 (78.3)	1369 (78.3)
Hispanic	104 (3.0)	52 (3.0)	52 (3.0)
Non-Hispanic Black	548 (15.7)	274 (15.7)	274 (15.7)
Other or Unk	106 (3.0)	53 (3.0)	53 (3.0)
Rurality[a] (%)			
Rural	1012 (28.9)	506 (28.9)	506 (28.9)
Urban	2474 (70.8)	1237 (70.8)	1237 (70.8)
Unknown	10 (0.3)	5 (0.3)	5 (0.3)
Region[b] (%)			
North Atlantic	792 (22.7)	396 (22.7)	396 (22.7)
Southeast	776 (22.2)	388 (22.2)	388 (22.2)
Midwest	924 (26.4)	462 (26.4)	462 (26.4)
Continental	450 (12.9)	225 (12.9)	225 (12.9)
Pacific	554 (15.8)	277 (15.8)	277 (15.8)
Charlson Score (%)			
0-2	2523 (72.2)	1299 (74.3)	1224 (70.0)
3-4	745 (21.3)	342 (19.6)	403 (23.1)
5+	228 (6.5)	107 (6.1)	121 (6.9)
Vaccine Type[c] (%)			
Moderna	-	-	907 (51.9)
Pfizer	-	-	841 (48.1)
Unknown	-	-	0 (0.0)
Cancer type[e] (%)			
Prostate	1358 (38.8)	679 (38.8)	679 (38.8)
SCCHN/CSCC/skin	514 (14.7)	257 (14.7)	257 (14.7)
Lung	498 (14.2)	249 (14.2)	249 (14.2)
Urothelial	208 (5.9)	104 (5.9)	104 (5.9)
Leukemia/MDS/MF	190 (5.4)	95 (5.4)	95 (5.4)
Lymphoma	132 (3.8)	66 (3.8)	66 (3.8)
HCC	112 (3.2)	56 (3.2)	56 (3.2)
Multiple myeloma	96 (2.7)	48 (2.7)	48 (2.7)
Colorectal	72 (2.1)	36 (2.1)	36 (2.1)
Esophagus/gastric	60 (1.7)	30 (1.7)	30 (1.7)
Other/unknown/lesser	256 (7.3)	128 (7.3)	128 (7.3)

© 2021 Wu JTY et al. *JAMA Oncology.*

[a] Defined based on patient home address.
[b] Defined based on VA station at which patient received cancer care.
[c] Vaccination type only shown for overall and vaccinated cohort, even though patients in the unvaccinated cohort can be subsequently vaccinated after matching, as those vaccinations are already tabulated in the vaccinated cohort.
[d] Systemic therapy received prior to vaccination (if in the vaccinated cohort) or prior to entry date (if in the unvaccinated cohort) with timing determined by the last dose of systemic therapy received. Different treatment types defined as detailed in eMethods.
[e] Cancer type determined by ICD code(s) associated with cancer-directed systemic treatment as detailed in eMethods. Other/unknown/lesser includes cancer of unknown primary, cancer types not included in a predefined category, and cancer types represented by 50 patients or less. CSCC = cutaneous squamous cell carcinoma; HCC = hepatocellular carcinoma; MF = myelofibrosis; MDS = myelodysplastic syndrome; SCCHN = squamous cell head and neck cancer.

© 2021 Wu JTY et al. *JAMA Oncology.*

eTable 10. Demographic and Clinical Characteristics of Matched Vaccinated and Unvaccinated VA Cancer Patients on Current Chemotherapy-containing Treatment (<3 Months).

Baseline Covariates	Overall (N=8452)	Unvaccinated (N=4226)	Vaccinated (N=4226)
Age (median [IQR])	72.88 [67.92, 76.49]	72.26 [66.80, 75.64]	73.35 [68.90, 77.34]
Age (%)			
<50	169 (2.0)	106 (2.5)	63 (1.5)
50-59	520 (6.2)	314 (7.4)	206 (4.9)
60-69	2019 (23.9)	1094 (25.9)	925 (21.9)
70-79	4555 (53.9)	2227 (52.7)	2328 (55.1)
>=80	1189 (14.1)	485 (11.5)	704 (16.7)
Gender = M (%)	8048 (95.2)	3996 (94.6)	4052 (95.9)
Race/Ethnicity (%)			
Non-Hispanic White	6702 (79.3)	3351 (79.3)	3351 (79.3)
Hispanic	248 (2.9)	124 (2.9)	124 (2.9)
Non-Hispanic Black	1272 (15.0)	636 (15.0)	636 (15.0)
Other or Unk	230 (2.7)	115 (2.7)	115 (2.7)
Rurality[a] (%)			
Rural	2480 (29.3)	1240 (29.3)	1240 (29.3)
Urban	5966 (70.6)	2983 (70.6)	2983 (70.6)
Unknown	6 (0.1)	3 (0.1)	3 (0.1)
Region[b] (%)			
North Atlantic	2010 (23.8)	1005 (23.8)	1005 (23.8)
Southeast	1740 (20.6)	870 (20.6)	870 (20.6)
Midwest	2236 (26.5)	1118 (26.5)	1118 (26.5)
Continental	1040 (12.3)	520 (12.3)	520 (12.3)
Pacific	1426 (16.9)	713 (16.9)	713 (16.9)
Charlson Score (%)			
0-2	4595 (54.4)	2395 (56.7)	2200 (52.1)
3-4	2722 (32.2)	1307 (30.9)	1415 (33.5)
5+	1135 (13.4)	524 (12.4)	611 (14.5)
Vaccine Type[c] (%)			
Moderna	-	-	2102 (49.7)
Pfizer	-	-	2122 (50.2)
Unknown	-	-	2 (0.0)
Cancer type[d] (%)			
Lung	1298 (15.4)	649 (15.4)	649 (15.4)
SCCHN/CSCC/skin	1272 (15.0)	636 (15.0)	636 (15.0)
Prostate	996 (11.8)	498 (11.8)	498 (11.8)
Colorectal	898 (10.6)	449 (10.6)	449 (10.6)
Urothelial	748 (8.8)	374 (8.8)	374 (8.8)
Essential thrombocythemia	524 (6.2)	262 (6.2)	262 (6.2)
Leukemia/MDS/MF	424 (5.0)	212 (5.0)	212 (5.0)
Lymphoma	364 (4.3)	182 (4.3)	182 (4.3)
Polycythemia vera	344 (4.1)	172 (4.1)	172 (4.1)
Other GI	226 (2.7)	113 (2.7)	113 (2.7)

© 2021 Wu JTY et al. *JAMA Oncology.*

Esophagus/gastric	216 (2.6)	108 (2.6)	108 (2.6)
HCC	188 (2.2)	94 (2.2)	94 (2.2)
Multiple myeloma	124 (1.5)	62 (1.5)	62 (1.5)
Breast	86 (1.0)	43 (1.0)	43 (1.0)
Neuroendocrine	82 (1.0)	41 (1.0)	41 (1.0)
Melanoma	56 (0.7)	28 (0.7)	28 (0.7)
Other/unknown/lesser	606 (7.2)	303 (7.2)	303 (7.2)

[a] Defined based on patient home address.
[b] Defined based on VA station at which patient received cancer care.
[c] Vaccination type only shown for overall and vaccinated cohort, even though patients in the unvaccinated cohort can be subsequently vaccinated after matching, as those vaccinations are already tabulated in the vaccinated cohort.
[d] Cancer type determined by ICD code(s) associated with cancer-directed systemic treatment as detailed in eMethods. Other/unknown/lesser includes cancer of unknown primary, cancer types not included in a predefined category, and cancer types represented by 50 patients or less. CSCC = cutaneous squamous cell carcinoma; HCC = hepatocellular carcinoma; MF = myelofibrosis; MDS = myelodysplastic syndrome; SCCHN = squamous cell head and neck cancer.

© 2021 Wu JTY et al. *JAMA Oncology.*

eTable 11. Demographic and Clinical Characteristics of Matched Vaccinated and Unvaccinated VA Cancer Patients on Current Targeted Treatment (<3 Months).

Baseline Covariates	Overall (N=8584)	Unvaccinated (N=4292)	Vaccinated (N=4292)
Age (median [IQR])	73.37 [68.86, 77.31]	72.97 [67.92, 76.48]	73.79 [69.66, 78.08]
Age (%)			
<50	197 (2.3)	128 (3.0)	69 (1.6)
50-59	555 (6.5)	325 (7.6)	230 (5.4)
60-69	1736 (20.2)	899 (20.9)	837 (19.5)
70-79	4670 (54.4)	2319 (54.0)	2351 (54.8)
>=80	1426 (16.6)	621 (14.5)	805 (18.8)
Gender = M (%)	8240 (96.0)	4089 (95.3)	4151 (96.7)
Race/Ethnicity (%)			
Non-Hispanic White	6572 (76.6)	3286 (76.6)	3286 (76.6)
Hispanic	278 (3.2)	139 (3.2)	139 (3.2)
Non-Hispanic Black	1442 (16.8)	721 (16.8)	721 (16.8)
Other or Unk	292 (3.4)	146 (3.4)	146 (3.4)
Rurality[a] (%)			
Rural	2640 (30.8)	1320 (30.8)	1320 (30.8)
Urban	5922 (69.0)	2961 (69.0)	2961 (69.0)
Unknown	22 (0.3)	11 (0.3)	11 (0.3)
Region[b] (%)			
North Atlantic	1856 (21.6)	928 (21.6)	928 (21.6)
Southeast	1852 (21.6)	926 (21.6)	926 (21.6)
Midwest	2140 (24.9)	1070 (24.9)	1070 (24.9)
Continental	1234 (14.4)	617 (14.4)	617 (14.4)
Pacific	1502 (17.5)	751 (17.5)	751 (17.5)
Charlson Score (%)			
0-2	5297 (61.7)	2764 (64.4)	2533 (59.0)
3-4	2392 (27.9)	1151 (26.8)	1241 (28.9)
5+	895 (10.4)	377 (8.8)	518 (12.1)
Vaccine Type[c] (%)			
Moderna	-	-	2320 (54.1)
Pfizer	-	-	1970 (45.9)
Unknown	-	-	2 (0.0)
Cancer type[d] (%)			
Leukemia/MDS/MF	2926 (34.1)	1463 (34.1)	1463 (34.1)
Multiple myeloma	2690 (31.3)	1345 (31.3)	1345 (31.3)
SCCHN/CSCC/skin	1020 (11.9)	510 (11.9)	510 (11.9)
Lymphoma	594 (6.9)	297 (6.9)	297 (6.9)
Prostate	212 (2.5)	106 (2.5)	106 (2.5)
RCC	170 (2.0)	85 (2.0)	85 (2.0)
Lung	158 (1.8)	79 (1.8)	79 (1.8)
Esophagus/gastric	104 (1.2)	52 (1.2)	52 (1.2)
Colorectal	84 (1.0)	42 (1.0)	42 (1.0)
Breast	82 (1.0)	41 (1.0)	41 (1.0)
HCC	76 (0.9)	38 (0.9)	38 (0.9)

© 2021 Wu JTY et al. *JAMA Oncology.*

Neuroendocrine	54 (0.6)	27 (0.6)	27 (0.6)
Other/unknown/lesser	414 (4.8)	207 (4.8)	207 (4.8)

[a] Defined based on patient home address.
[b] Defined based on VA station at which patient received cancer care.
[c] Vaccination type only shown for overall and vaccinated cohort, even though patients in the unvaccinated cohort can be subsequently vaccinated after matching, as those vaccinations are already tabulated in the vaccinated cohort.
[d] Cancer type determined by ICD code(s) associated with cancer-directed systemic treatment as detailed in eMethods. Other/unknown/lesser includes cancer of unknown primary, cancer types not included in a predefined category, and cancer types represented by 50 patients or less. CSCC = cutaneous squamous cell carcinoma; HCC = hepatocellular carcinoma; MF = myelofibrosis; MDS = myelodysplastic syndrome; RCC = renal cell carcinoma; SCCHN = squamous cell head and neck cancer.

© 2021 Wu JTY et al. *JAMA Oncology.*

eTable 12. Demographic and Clinical Characteristics of Matched Vaccinated and Unvaccinated VA Cancer Patients on Current Endocrine Treatment (<3 Months).

Baseline Covariates	Overall (N=15514)	Unvaccinated (N=7757)	Vaccinated (N=7757)
Age (median [IQR])	74.53 [70.71, 80.80]	74.22 [70.28, 80.43]	74.92 [71.18, 81.25]
Age (%)			
<50	166 (1.1)	102 (1.3)	64 (0.8)
50-59	559 (3.6)	304 (3.9)	255 (3.3)
60-69	2705 (17.4)	1450 (18.7)	1255 (16.2)
70-79	7893 (50.9)	3883 (50.1)	4010 (51.7)
>=80	4191 (27.0)	2018 (26.0)	2173 (28.0)
Gender = M (%)	14540 (93.7)	7260 (93.6)	7280 (93.9)
Race/Ethnicity (%)			
Non-Hispanic White	9406 (60.6)	4703 (60.6)	4703 (60.6)
Hispanic	866 (5.6)	433 (5.6)	433 (5.6)
Non-Hispanic Black	4418 (28.5)	2209 (28.5)	2209 (28.5)
Other or Unk	824 (5.3)	412 (5.3)	412 (5.3)
Rurality[a] (%)			
Rural	4584 (29.5)	2292 (29.5)	2292 (29.5)
Urban	10808 (69.7)	5404 (69.7)	5404 (69.7)
Unknown	122 (0.8)	61 (0.8)	61 (0.8)
Region[b] (%)			
North Atlantic	3672 (23.7)	1836 (23.7)	1836 (23.7)
Southeast	3534 (22.8)	1767 (22.8)	1767 (22.8)
Midwest	3786 (24.4)	1893 (24.4)	1893 (24.4)
Continental	1906 (12.3)	953 (12.3)	953 (12.3)
Pacific	2616 (16.9)	1308 (16.9)	1308 (16.9)
Charlson Score (%)			
0-2	9134 (58.9)	4732 (61.0)	4402 (56.7)
3-4	4748 (30.6)	2288 (29.5)	2460 (31.7)
5+	1632 (10.5)	737 (9.5)	895 (11.5)
Vaccine Type[c] (%)			
Moderna	-	-	4331 (55.8)
Pfizer	-	-	3423 (44.1)
Unknown	-	-	3 (0.0)
Cancer type[d] (%)			
Prostate	14180 (91.4)	7090 (91.4)	7090 (91.4)
Breast	1060 (6.8)	530 (6.8)	530 (6.8)
Neuroendocrine	130 (0.8)	65 (0.8)	65 (0.8)
Other/unknown/lesser	144 (0.9)	72 (0.9)	72 (0.9)

[a] Defined based on patient home address.
[b] Defined based on VA station at which patient received cancer care.

© 2021 Wu JTY et al. *JAMA Oncology.*

[c] Vaccination type only shown for overall and vaccinated cohort, even though patients in the unvaccinated cohort can be subsequently vaccinated after matching, as those vaccinations are already tabulated in the vaccinated cohort.
[d] Cancer type determined by ICD code(s) associated with cancer-directed systemic treatment as detailed in eMethods. Other/unknown/lesser includes cancer of unknown primary, cancer types not included in a predefined category, and cancer types represented by 50 patients or less.

© 2021 Wu JTY et al. *JAMA Oncology.*

eTable 13. Cancer-directed Systemic Therapies by Treatment Type.

Treatment	Type
abemaciclib	Targeted therapy
abiraterone	Endocrine therapy
abvd	Chemotherapy
abve	Chemotherapy
abve-pc	Chemotherapy
ac	Chemotherapy
ac-t	Chemotherapy
acalabrutinib	Targeted therapy
ade	Chemotherapy
ado trastuzumab	Targeted therapy
afatinib	Targeted therapy
aldesleukin	Immunotherapy
alectinib	Targeted therapy
alemtuzumab	Targeted therapy
aminolevulinic	Targeted therapy
anastrozole	Endocrine therapy
apalutamide	Endocrine therapy
arsenic	Chemotherapy
asparaginase erwinia chrysanthemi	Chemotherapy
atezolizumab	Immunotherapy
avelumab	Immunotherapy
axicabtagene	Targeted therapy
axitinib	Targeted therapy
azacitidine	Chemotherapy
beacopp	Chemotherapy
belinostat	Targeted therapy
bendamustine	Chemotherapy
bep	Chemotherapy
bevacizumab	Targeted therapy
bexarotene	Targeted therapy
bicalutamide	Endocrine therapy
binimetinib	Targeted therapy
bleomycin	Chemotherapy
blinatumomab	Immunotherapy
bortezomib	Targeted therapy
bosutinib	Targeted therapy
brentuximab	Targeted therapy
brigatinib	Targeted therapy
bumel	Chemotherapy

© 2021 Wu JTY et al. *JAMA Oncology.*

busulfan	Chemotherapy
cabazitaxel	Chemotherapy
cabozantinib	Targeted therapy
caf	Chemotherapy
calaspargase	Chemotherapy
capecitabine	Chemotherapy
caplacizumab	Targeted therapy
capox	Chemotherapy
carboplatin	Chemotherapy
carboplatin-taxol	Chemotherapy
carfilzomib	Targeted therapy
carmustine	Chemotherapy
carmustine	Chemotherapy
cem	Chemotherapy
cemiplimab	Immunotherapy
ceritinib	Targeted therapy
cetuximab	Targeted therapy
cev	Chemotherapy
chlorambucil	Chemotherapy
chlorambucil-prednisone	Chemotherapy
chop	Chemotherapy
cisplatin	Chemotherapy
cladribine	Chemotherapy
clofarabine	Chemotherapy
cmf	Chemotherapy
cobimetinib	Targeted therapy
copanlisib	Targeted therapy
copdac	Chemotherapy
copp	Chemotherapy
copp-abv	Chemotherapy
crizotinib	Targeted therapy
cvp	Chemotherapy
cyclophosphamide	Chemotherapy
cytarabine	Chemotherapy
cytarabine	Chemotherapy
dabrafenib	Targeted therapy
dacarbazine	Chemotherapy
dacomitinib	Targeted therapy
dactinomycin	Chemotherapy
daratumumab	Targeted therapy
dasatinib	Targeted therapy
daunorubicin	Chemotherapy

© 2021 Wu JTY et al. *JAMA Oncology.*

daunorubicin cytarabine	Chemotherapy
decitabine	Chemotherapy
degarelix	Endocrine therapy
denileukin	Targeted therapy
dinutuximab	Targeted therapy
docetaxel	Chemotherapy
doxorubicin	Chemotherapy
doxorubicin lipo	Chemotherapy
durvalumab	Immunotherapy
duvelisib	Targeted therapy
elotuzumab	Targeted therapy
emapalumab	Targeted therapy
enasidenib	Targeted therapy
encorafenib	Targeted therapy
enzalutamide	Endocrine therapy
epirubicin	Chemotherapy
epoch	Chemotherapy
erdafitinib	Targeted therapy
eribulin	Chemotherapy
erlotinib	Targeted therapy
etoposide	Chemotherapy
etoposide	Chemotherapy
everolimus	Targeted therapy
exemestane	Endocrine therapy
fec	Chemotherapy
fludarabine	Chemotherapy
fluorouracil inj	Chemotherapy
fluorouracil top	Chemotherapy
flutamide	Endocrine therapy
folfiri	Chemotherapy
folfiri-bevacizumab	Chemotherapy
folfiri-cetuximab	Chemotherapy
folfirinox	Chemotherapy
folfox	Chemotherapy
fostamatinib	Targeted therapy
fu-lv	Chemotherapy
fulvestrant	Endocrine therapy
gefitinib	Targeted therapy
gemcitabine	Chemotherapy
gemcitabine-cisplatin	Chemotherapy
gemcitabine-oxaliplatin	Chemotherapy
gemtuzumab	Targeted therapy

© 2021 Wu JTY et al. *JAMA Oncology.*

gilteritinib	Targeted therapy
glasdegib	Targeted therapy
goserelin	Endocrine therapy
hvp	Chemotherapy
hydroxyurea	Chemotherapy
hyper-cvad	Chemotherapy
ibritumomab	Targeted therapy
ibrutinib	Targeted therapy
ice	Chemotherapy
idarubicin	Chemotherapy
idelalisib	Targeted therapy
ifosfamide	Chemotherapy
imatinib	Targeted therapy
imiquimod	Targeted therapy
inotuzumab	Targeted therapy
interferon alfa	Immunotherapy
interferon alfa-2b	Immunotherapy
iobenguane i 131	Targeted therapy
ipilimumab	Immunotherapy
irinotecan	Chemotherapy
irinotecan liposome	Chemotherapy
ivosidenib	Targeted therapy
ixabepilone	Chemotherapy
ixazomib	Targeted therapy
jeb	Chemotherapy
lanreotide	Endocrine therapy
lapatinib	Targeted therapy
larotrectinib	Targeted therapy
lenalidomide	Targeted therapy
lenvatinib	Targeted therapy
letrozole	Endocrine therapy
leuprolide	Endocrine therapy
lomustine	Chemotherapy
lorlatinib	Targeted therapy
lu 177-dotatate	Targeted therapy
lutetium lu 177-dotatate	Targeted therapy
mechlorethamine	Chemotherapy
melphalan	Chemotherapy
melphalan	Chemotherapy
mercaptopurine	Chemotherapy
methotrexate	Chemotherapy
midostaurin	Targeted therapy

© 2021 Wu JTY et al. *JAMA Oncology.*

mitomycin c	Chemotherapy
mitoxantrone	Chemotherapy
mogamulizumab	Targeted therapy
moxetumomab	Targeted therapy
mvac	Chemotherapy
necitumumab	Targeted therapy
nelarabine	Chemotherapy
neratinib	Targeted therapy
nilotinib	Targeted therapy
nilutamide	Endocrine therapy
niraparib	Targeted therapy
nivolumab	Immunotherapy
obinutuzumab	Targeted therapy
oepa	Chemotherapy
ofatumumab	Targeted therapy
off	Chemotherapy
olaparib	Targeted therapy
olaratumab	Targeted therapy
omacetaxine	Targeted therapy
oppa	Chemotherapy
osimertinib	Targeted therapy
oxaliplatin	Chemotherapy
paclitaxel	Chemotherapy
paclitaxel nab	Chemotherapy
pad	Chemotherapy
palbociclib	Targeted therapy
panitumumab	Targeted therapy
panobinostat	Targeted therapy
pazopanib	Targeted therapy
pcv	Chemotherapy
peb	Chemotherapy
pegaspargase	Chemotherapy
peginterferon alfa	Immunotherapy
pembrolizumab	Immunotherapy
pemetrexed	Chemotherapy
pertuzumab	Targeted therapy
pomalidomide	Targeted therapy
ponatinib	Targeted therapy
pralatrexate	Chemotherapy
procarbazine	Chemotherapy
r-chop	Chemotherapy
r-cvp	Chemotherapy

© 2021 Wu JTY et al. *JAMA Oncology.*

r-epoch	Chemotherapy
r-ice	Chemotherapy
radium 223	Targeted therapy
raloxifene	Endocrine therapy
ramucirumab	Targeted therapy
ravulizumab	Targeted therapy
regorafenib	Targeted therapy
ribociclib	Targeted therapy
rituximab	Targeted therapy
rituximab and hyaluronidase human	Targeted therapy
romidepsin	Targeted therapy
rucaparib	Targeted therapy
ruxolitinib	Targeted therapy
siltuximab	Targeted therapy
sipuleucel	Immunotherapy
sonidegib	Targeted therapy
sorafenib	Targeted therapy
stanford v	Chemotherapy
sunitinib	Targeted therapy
tac	Chemotherapy
tagraxofusp	Targeted therapy
talazoparib	Targeted therapy
talimogene	Immunotherapy
tamoxifen	Endocrine therapy
temozolomide	Chemotherapy
temsirolimus	Targeted therapy
thalidomide	Targeted therapy
thioguanine	Chemotherapy
thiotepa	Chemotherapy
tisagenlecleucel	Immunotherapy
topotecan	Chemotherapy
toremifene	Endocrine therapy
tpf	Chemotherapy
trabectedin	Chemotherapy
trametinib	Targeted therapy
trastuzumab	Targeted therapy
trastuzumab and hyaluronidase-oysk	Targeted therapy
trifluridine and tipiracil hydrochloride	Chemotherapy
vac	Chemotherapy
valrubicin	Chemotherapy

© 2021 Wu JTY et al. *JAMA Oncology.*

vamp	Chemotherapy
vandetanib	Targeted therapy
veip	Chemotherapy
vemurafenib	Targeted therapy
venetoclax	Targeted therapy
vinblastine	Chemotherapy
vincristine	Chemotherapy
vincristine	Chemotherapy
vinorelbine	Chemotherapy
vip	Chemotherapy
vismodegib	Targeted therapy
vorinostat	Targeted therapy
xeliri	Chemotherapy
xelox	Chemotherapy
ziv aflibercept	Targeted therapy

© 2021 Wu JTY et al. *JAMA Oncology.*
